# Evaluating the collection, comparability and findings of six global surgery indicators

**DOI:** 10.1002/bjs.11061

**Published:** 2018-12-20

**Authors:** H. Holmer, A. Bekele, L. Hagander, E. M. Harrison, P. Kamali, J. S. Ng‐Kamstra, M. A. Khan, L. Knowlton, A. J. M. Leather, I. H. Marks, J. G. Meara, M. G. Shrime, M. Smith, K. Søreide, T. G. Weiser, J. Davies

**Affiliations:** ^1^ WHO Collaborating Centre for Surgery and Public Health, Department of Clinical Sciences Lund, Faculty of Medicine, Lund University Lund Sweden; ^2^ Department of Paediatric Surgery, Skåne University Hospital Children's Hospital Lund Sweden; ^3^ Karolinska University Hospital Solna, Stockholm Sweden; ^4^ Department of Surgery, School of Medicine, Addis Ababa University Addis Ababa Ethiopia; ^5^ University of Global Health Equity Kigali Rwanda; ^6^ Department of Clinical Surgery, Royal Infirmary of Edinburgh and Surgical Informatics, Centre for Medical Informatics, Usher Institute, University of Edinburgh Edinburgh UK; ^7^ King's Centre for Global Health and Health Partnerships, School of Population Health and Environmental Sciences, Faculty of Life Sciences and Medicine, King's College London London UK; ^8^ Charing Cross Hospital, Imperial College Healthcare NHS Trust London UK; ^9^ Institute for Applied Health Research, University of Birmingham Birmingham UK; ^10^ Division of Plastic and Reconstructive Surgery, Medisch Spectrum Twente Enschede the Netherlands; ^11^ InciSioN, International Student Surgical Network Leuven Belgium; ^12^ Department of Critical Care Medicine, University of Calgary, Calgary Alberta Canada; ^13^ CMH Lahore Medical College and Institute of Dentistry Lahore Pakistan; ^14^ Department of Surgery, Stanford University Stanford, California USA; ^15^ Department of Global Health and Social Medicine, Harvard Medical School Boston USA; ^16^ Department of Plastic and Oral Surgery, Boston Children's Hospital Boston USA; ^17^ Center for Global Surgery Evaluation, Massachusetts Eye and Ear Infirmary Boston USA; ^18^ Department of Surgery, Faculty of Health Sciences, University of the Witwatersrand Johannesburg South Africa; ^19^ Department of General Surgery, Chris Hani Baragwaneth Academic Hospital Johannesburg South Africa; ^20^ MRC/Wits Rural Public Health and Health Transitions Research Unit, School of Public Health, University of Witwatersrand Parktown South Africa; ^21^ Department of Gastrointestinal Surgery, Stavanger University Hospital Stavanger Norway; ^22^ Department of Clinical Medicine, University of Bergen Bergen Norway

## Abstract

**Background:**

In 2015, six indicators were proposed to evaluate global progress towards access to safe, affordable and timely surgical and anaesthesia care. Although some have been adopted as core global health indicators, none has been evaluated systematically. The aims of this study were to assess the availability, comparability and utility of the indicators, and to present available data and updated estimates.

**Methods:**

Nationally representative data were compiled for all World Health Organization (WHO) member states from 2010 to 2016 through contacts with official bodies and review of the published and grey literature, and available databases. Availability, comparability and utility were assessed for each indicator: access to timely essential surgery, specialist surgical workforce density, surgical volume, perioperative mortality, and protection against impoverishing and catastrophic expenditure. Where feasible, imputation models were developed to generate global estimates.

**Results:**

Of all WHO member states, 19 had data on the proportion of the population within 2h of a surgical facility, 154 had data on workforce density, 72 reported number of procedures, and nine had perioperative mortality data, but none could report data on catastrophic or impoverishing expenditure. Comparability and utility were variable, and largely dependent on different definitions used. There were sufficient data to estimate that worldwide, in 2015, there were 2 038 947 (i.q.r. 1 884 916–2 281 776) surgeons, obstetricians and anaesthetists, and 266·1 (95 per cent c.i. 220·1 to 344·4) million operations performed.

**Conclusion:**

Surgical and anaesthesia indicators are increasingly being adopted by the global health community, but data availability remains low. Comparability and utility for all indicators require further resolution.

## Graphical abstract

This paper examines the availability, comparability and utility of six global surgery indicators. Surgical and anaesthesia indicators are increasingly being adopted by the global health community, but data availability remains low. Comparability and utility for all indicators require further resolution.

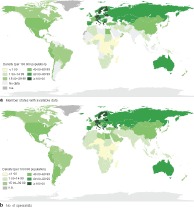

An update, and a plea for improvement

## Introduction

Surgical, obstetric and anaesthesia care (hereafter referred to as surgical care) are core components of a functional healthcare system, yet improving access to these essential services has not been a priority in global health. Recognizing the need to improve access to safe, affordable and timely surgical care, the Lancet Commission on Global Surgery^1^ brought together providers, academics and policymakers from 110 countries to describe the current state of surgery globally and chart a route for improvement. In the Commission report^2^ were two key recommendations: for countries to develop National Surgical, Obstetric and Anaesthesia Plans, and for stakeholders to track and report core surgical indicators to assess progress, identify opportunities to improve access and quality of care, and advocate for those in need^2^, ^3^.

As surgery is a treatment modality for diverse conditions, its effective assessment demands indicators that capture health system dimensions of capacity, service delivery and outcomes. The Commission identified six core indicators – with targets – for monitoring access to, delivery of, and outcomes from surgical care (*Table* 1).

**Table 1:** Six indicators to assess access to safe, affordable and timely surgical and anaesthesia care^2^

Indicator	Definition
Indicator 1: access to timely essential surgery	Proportion of the population living within 2h of a facility able to provide three critically essential procedures – laparotomy, caesarean delivery and fixation of an open fracture – called the bellwether procedures, as reflective of a facility's ability to provide most other essential surgical procedures. The target was 80% of the population within 2h of a facility able to provide the bellwether procedures by 2030
Indicator 2: specialist surgical workforce density	Number of physician specialist surgeons, obstetricians and anaesthetists actively working per 100 000 people. The target was a minimum of 20 providers per 100 000 people in 2030, based on the provider density associated with declining maternal mortality
Indicator 3: surgical volume	Total number of operations, defined as the incision, excision, or manipulation of tissue that needs regional or general anaesthesia, or profound sedation to control pain^4^, carried out per 100 000 people per year. The target was 5000 operations per 100 000 population, determined using three strategies to triangulate the number of operations needed
Indicator 4: perioperative mortality	Number of in‐hospital deaths following any procedure done in an operating theatre, divided by the total number of procedures, presented as percentage (perioperative mortality rate). In‐hospital mortality was chosen over 30‐day mortality to enhance feasibility globally. There was no target set, but a recommendation that by 2020 at least 80% of countries should track data on perioperative mortality, and 100% by 2030
Indicator 5–6: protection against impoverishing and catastrophic expenditure	Proportion of the population who, if they needed a surgical operation, would be protected against impoverishing (pushing the household below the poverty level), or catastrophic (equalling more than 40% of household income, excluding subsistence needs) expenditure. The target selected was 100% protection from impoverishing or catastrophic expenditure related to accessing surgical and anaesthesia care by 2030

Some of these indicators have now been included among the WHO 100 Core Global Health Indicators^5^ and the World Bank's World Development Indicators^6^, ^7^. WHO member states have called repeatedly for increased data collection through the World Health Assembly in support of the Sustainable Development Goals^8^, ^9^, ^10^. In proposing these indicators, there was recognition of the need to evaluate them after a period of data collection to ensure that they are useful and valuable for improving access to safe, affordable and timely surgical care globally. The objectives of this study were twofold. The first was to review the indicators using the framework of availability, comparability and utility. Availability is the number of countries with nationally representative data, comparability is the number of definitions used for each indicator, and utility is a discursive appraisal of whether the indicator as collected and reported fulfils its intended purpose. The second objective was to report the numerical results of each indicator for each country using actual data or derived estimates, where possible.

## Methods

### Data collection

During 2015 and 2016, nationally representative data from 194 WHO member states were collected through direct contact with official bodies and by reviewing the published and grey literature, as well as available databases. Request letters (in English, Arabic, Chinese, French and Spanish) were sent by e‐mail to Ministries of Health with an available address. Where they did not respond or were not able to provide the requested data, or where no e‐mail was available, United Nations and WHO offices, relevant statistical bodies, professional societies, and individual clinicians and academics were contacted. Up to three follow‐up e‐mails were sent; where there was no response, contact by telephone was attempted.

In addition, official websites of Ministries of Health, statistical bodies and professional societies, and public databases from the European Commission, Organisation for Economic Co‐operation and Development (OECD), WHO and the World Bank were searched to identify data on the six indicators. Finally, PubMed and MEDLINE were searched using each country's name along with the following keywords and phrases: ‘surgery’, ‘procedures’, ‘national surgical volume’, ‘national surgical rate’, ‘access to surgical care’, ‘surgeons’, ‘anaesthetists’, ‘anaesthesiologists’ and ‘obstetricians’. Referenced publications were also reviewed and included when relevant.

Health and development indicators were retrieved from the World Bank's World Development Indicators^6^ and the from the WHO Global Health Observatory^11^.

### Inclusion criteria

Only nationally representative data aligning with the Commission's indicator definitions from the 194 WHO member states for the years 2010–2016 were included.

### Data review and selection

In reviewing availability, comparability and utility of indicators, all data points that met inclusion criteria were considered. Alternative indicator definitions were also recorded. When presenting country‐level results by indicator, and for modelling purposes, the most recent and reliable data point was selected, giving preference to data reported by Ministries of Health and other official national bodies.

Median (i.q.r.) values are used to describe indicators with a non‐normal distribution; results are shown by World Bank income category^6^ and WHO region^12^. Statistical analysis was performed in R (R Foundation for Statistical Computing, Vienna, Austria).

### Deriving each indicator

#### *Indicator 1: access to timely essential surgery*

Global Positioning System coordinates were sought for hospitals listed as performing laparotomy, caesarean section and open fracture repair (bellwether procedures^2^). Whether these procedures were performed was not validated further. A previously published methodology for calculating the proportion of the population within 2 hours of these facilities was employed^13^. For countries with data, facility coordinates were plotted using Redivis (Redivis, Palo Alto, California, USA)^14^, an online data visualization platform. A 2h catchment area was calculated based on road network data from OpenStreetMaps^15^ by assigning approximate travel speeds of 100, 50 and 30 km/h based on the type of roadway. Areas without roadways were assigned an average walking speed of 5 km/h. The proportion of the total population in 2013 (the latest year for which data were available in 2016) within 2h was calculated using WorldPop^16^, a global population distribution database, and tabulated as a percentage.

#### *Indicator 2: specialist surgical workforce density*

Data were compiled on the total number of specialist physician surgeons, obstetricians and anaesthetists actively working by country. For countries with multiple data points, the most recent and reliable data point was selected based on a hierarchical approach (*Table* *S1*, supporting information). The number of providers in each of the three categories was divided by the total country population in the corresponding year to calculate number of providers per 100 000 people, presented separately and as the sum of all three provider categories.

The number of providers was estimated for countries without primary data using a previously employed methodology^17^ based on a set of 16 health system and development indicators (*Table S2*, supporting information). Multiple imputation was used to estimate missing data for 2015, generating 100 imputations to estimate the number of providers per 100 000 people. Due to the non‐normal distribution of imputed values, median and i.q.r. are used to describe the data.

#### *Indicator 3: surgical volume*

Data were compiled on the total number of procedures performed in an operating theatre per country. For countries with multiple data points, the most recent and reliable data point was selected based on a hierarchical approach (*Table S3*, supporting information). The total number of procedures was divided by total population for the corresponding year.

To estimate the number of procedures done in 2015, a model was developed based on the method proposed by Weiser and colleagues^18^. The Spearman correlation between surgical volume and five country‐level variables was analysed (total population, life expectancy, percentage urbanization, gross domestic product per capita and total health expenditure per capita), from which health expenditure was selected as the explanatory variable (*Table S4*, supporting information). If health expenditure data were not available for 2015, values were extrapolated from 2010–2014 by adjusting for inflation using the Consumer Price Index^19^. Countries without data during that time interval were excluded from the analysis. Matching health expenditure by country and year with available surgical volume data, spline models were built with zero, one, two and three inflection points. As these yielded similar results, the model with two inflection points was selected to align with the previous study^18^ for comparability. For countries with available surgical volume data from 2010–2014, the volume of surgery in 2015 was estimated using the spline model with two inflection points and treating 2015 volume of surgery as missing. Volume reported in 2016 was assumed to be equivalent to that in 2015 (the year for which health expenditure data were available). Using the resulting data set, missing surgical volume data were imputed for countries with no data available; 300 imputations were done to calculate national estimates and an estimated total global volume with 95 per cent confidence intervals.

#### *Indicator 4: perioperative mortality*

Data were sought on the number of deaths following surgery and volume of surgery for the corresponding year. Mortality data that pertained to only a subset of procedures or used estimated rather than empirical values were excluded. The definition of perioperative mortality used was also recorded. As the target for this indicator was for a country to collect data, only whether data were found and the definition used, not the exact perioperative mortality value by country, is presented here.

#### *Indicators 5–6: protection against impoverishing and catastrophic expenditure*

Data were sought on the cost of the bellwether procedures at first‐level and tertiary‐level hospitals within each country. It was intended to compute an update of previous analyses by Shrime and co‐workers^20^, ^21^, ^22^ regarding the proportion of the population protected against impoverishing and catastrophic expenditure.

#### *Assessment of utility*

The authors held one meeting in person to appraise the results and discuss the utility of each indicator in terms of fulfilment of its intended purpose. Further discussions were held via e‐mail and Skype until agreement was reached.

## Results

Summary data for all indicators are presented in *Table* 2.

**Table 2:** Findings by indicator related to availability and comparability

Indicator	Availability (no. of countries)	Comparability (list of definitions found)
Indicator 1: access to timely essential surgery	19	Hospitals providing bellwether procedures Any hospital type
Indicator 2: specialist surgical workforce density	154 (166 with data on any of the 3 categories)	All licensed specialist surgeons, anaesthetists and obstetricians* All licensed specialist surgeons, anaesthetists and obstetricians including trainees Surgical group of specialists (including surgeons, anaesthetists and emergency physicians)
Indicator 3: surgical volume	72	All procedures done in an operating theatre All inpatient procedures done in an operating theatre Specific set of procedures
Indicator 4: perioperative mortality	9 (28 using any definition of postoperative mortality)	30‐day postoperative mortality rate In‐hospital postoperative mortality rate Not specified
Indicator 5–6: protection against impoverishing and catastrophic expenditure	0 (186 modelled)	–

* This definition can be subdivided further depending on whether the source included ophthalmologists, maxillofacial surgeons, and ear, nose and throat specialists in the surgeon category; intensivists in the anaesthetics category; and gynaecologists in the obstetrician category.

### Indicator 1: access to timely essential surgery

**Figure 1:**
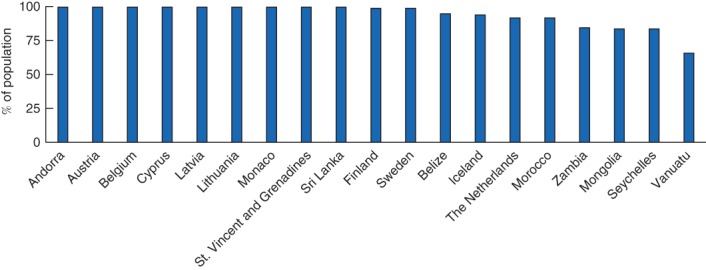
Proportion of population within 2h of a facility able to provide laparotomy, caesarean section and open fracture repair

Data were available from 12 countries for 2015 and nine countries for 2016. Two countries had data from both years. In total, 19 countries were included (*Fig*. 1), with a majority of data coming from Ministries of Health (*Table S5*, supporting information). There were 12 high‐income, two upper‐middle‐income and five lower‐middle‐income countries. No low‐income countries provided data. Only Vanuatu, a lower‐middle‐income country, fell below the 80 per cent target, and 12 of the 19 countries had 95 per cent or more of the population residing within 2h of a facility listed as performing the three bellwether procedures (*Fig*. 1).

Regarding utility, to be measurable this indicator requires identification of facilities capable of performing bellwether procedures, knowledge of local road speeds, and distribution of the population relative to facilities. The lack of precise definitions of two of the three bellwether procedures (laparotomy and open fracture repair), and the meaning of ‘capable’, provide challenges to both utility and comparability. There are further issues with estimating travel time to facilities as road speeds may vary depending on many factors. Finally, this indicator does not capture whether patients actually receive care, but simply whether they could travel to a facility where care is provided.

### Indicator 2: specialist surgical workforce density

**Figure 2:**
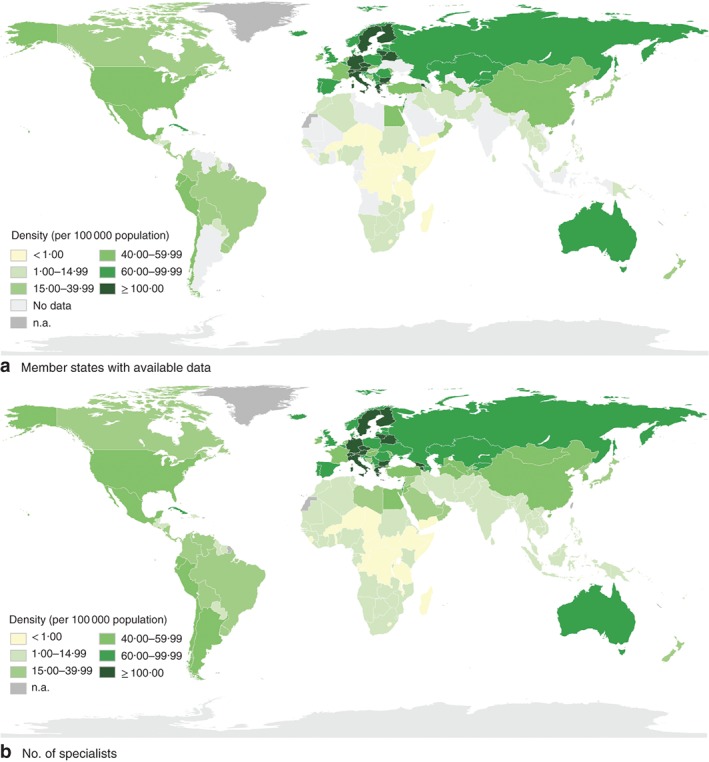
Specialist surgeons, obstetricians and anaesthetists per 100 000 people. **a** WHO member states with complete data on specialist surgeons, obstetricians and anaesthetists, 2010–2016, and **b** estimated number of specialist surgeons, obstetricians and anaesthetists per 100 000 people in WHO member states in 2015. n.a, Countries or territories that are not WHO members and therefore excluded from the data

A total of 3615 data points were found from 183 countries, including data from eight different but overlapping databases (*Tables S6* and *S7*, supporting information). Data were found on all three specialties from 154 WHO member states (*Fig*. 2 *a*), and data on one or two specialties were found from an additional 12 countries, together representing 86 per cent of all WHO member states and 74 per cent of their population. Considering comparability, three different general definitions were identified, in addition to local variations at the country level (*Table* 2; *Table S8*, supporting information), and as much as a 300 per cent difference in the numbers of providers between data sources from the same country and the same or adjacent years was noted. The two WHO European Regional Office databases^23^, ^24^ contained data on ‘Surgical group of specialists’, which includes emergency medicine and anaesthetists. These were excluded from global estimates of provider numbers, except where the number of emergency medicine and anaesthetic physicians could be identified separately and subtracted from the total number.

In countries with available data, the median number of specialist physician surgical, anaesthesia and obstetric providers was 68 (i.q.r. 49–90) per 100 000 in high‐income countries, 24 (11–55) per 100 000 in upper‐middle‐income countries, 4 (2–15) per 100 000 in lower‐middle‐income countries, and 0·7 (0·4–1·7) per 100 000 in low‐income countries (*Table S9*, supporting information). Based on estimates for 2015, there were 2 038 947 (i.q.r. 1 884 916–2 281 776) surgeons, obstetricians and anaesthetists globally in 2015, or 1 097 052 (1 007 340–1 235 323) surgeons, 576 749 (529 595–637 836) obstetricians and 365 146 (347 981–408 617) anaesthetists (*Fig*. 2 *b*; *Table S10*, supporting information); 100 of the 194 WHO member states were below the threshold of 20 providers per 100 000 people (*Tables S11* and *S12*, supporting information).

Concerning utility, the main limitation was the lack of comparability resulting from multiple definitions. Furthermore, this indicator has not been disaggregated to capture more granular aspects such as provider productivity, age, public or private sector activity, or geographical distribution. All are useful for national planning, although such granular data are less feasible to collect for global benchmarking. It was also noted that this metric does not capture non‐specialist providers (general physicians and non‐physicians), nor the supporting staff needed to provide surgical services; however, others^25^, ^26^, ^27^ are working to ensure that these groups are counted and recognized.

### Indicator 3: surgical volume

**Figure 3:**
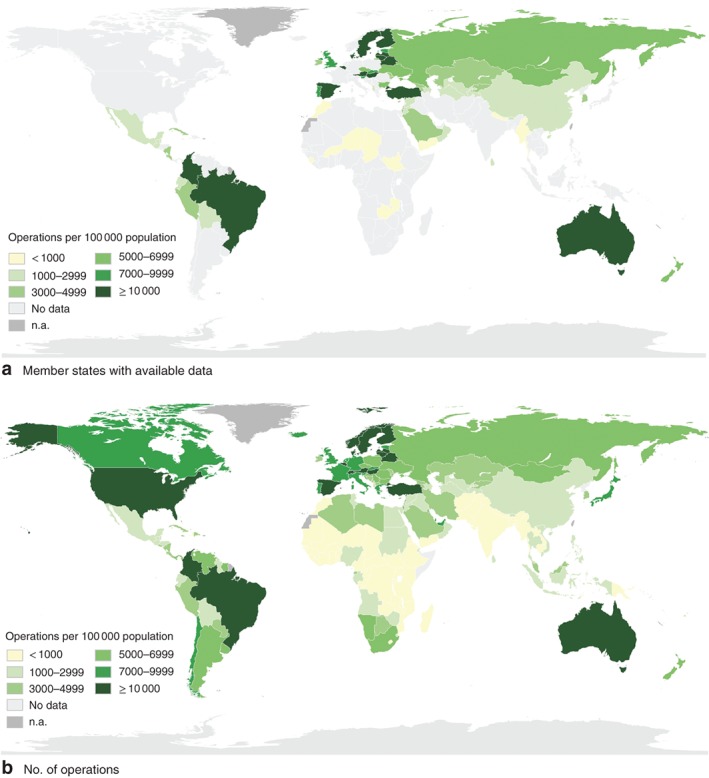
Annual number of surgical operations per 100 000 population. **a** WHO member states with available data on annual number of surgical operations, 2010–2016, and **b** estimated number of surgical operations per 100 000 population in WHO member states in 2015. n.a, Countries or territories that are not WHO members and therefore excluded from the data

A total of 1221 data points were found from 102 countries. After removing data that did not meet inclusion criteria, 72 countries remained, corresponding to 37 per cent of WHO member countries and 39 per cent of the global population (*Fig*. 3 *a*; *Table S13,* supporting information). There were five different but overlapping databases using three different definitions of surgical procedures (*Table* 2; *Tables S14 and S15,* supporting information). No data from the OECD and Eurostat databases were included as these sources did not present the total number of procedures but rather the number of procedures for specific sets of operations.

In countries with available data, the median number of operations was 7579 (i.q.r. 5014–10 891) per 100 000 people in high‐income countries, 3375 (2034–12 352) per 100 000 in upper‐middle‐income countries, 2445 (1012–4731) per 100 000 in lower‐middle‐income countries and 328 (231–513) per 100 000 in low‐income countries (*Table S16,* supporting information).

Of the five country variables tested against national surgical volume, health expenditure stood out as the most significantly correlated variable (Spearman's *r*_s_ = 0·724; *P* < 0·001) (*Table S4*, supporting information) and was used to develop the spline model employed to estimate the global volume of surgery (*Fig. S1*, supporting information). In 2015, an estimated 266·1 (95 per cent c.i. 220·1 to 344·4) million operations were done worldwide (*Fig*. 3 *b*; *Table S17*, supporting information). Somalia and Democratic People's Republic of Korea were excluded owing to lack of health expenditure data. Based on these estimates, 90 of the 194 WHO member states are below the Commission's recommended target of 5000 operations per 100 000 people (*Tables S18* and *S19*, supporting information).

Concerning utility, a major challenge is the numerous definitions of surgical procedure^18^, ^28^ as well as different procedure classification systems^29^, ^30^, ^31^. There are particular challenges capturing surgical volume in the private sector. It was also noted that the definition put forward by the Commission does not take into account differing country need^28^.

### Indicator 4: perioperative mortality

Nationwide data on number of deaths following operations were available from 28 WHO member states (*Table* 2; *Table S20*, supporting information). Concerning comparability, there were two different definitions used: nine countries relied exclusively on in‐hospital mortality, seven used 30‐day mortality and 12 provided no definition. Without information on case mix and preoperative patient risk, comparability would remain limited, and potential to game this indicator and achieve targets by doing low‐risk procedures on fit patients would be high. The utility of this metric for benchmarking is limited primarily by data availability; however, comparability would be likely to influence utility even if data were available more widely.

### Indicators 5–6: protection against impoverishing and catastrophic expenditure

No data determined to be nationally representative of the out‐of‐pocket cost of surgery were found, a prerequisite for calculating these metrics. Data were provided from five countries on the provider costs of surgery, which showed large variation between types of surgery and between sources (*Table S21*, supporting information)^20^, ^21^. Comparability and utility could not be assessed without available data; indeed, without any data, the indicators are of no use. However, in principle, these indicators are of huge value in assessing progress towards universal health coverage.

Unfortunately, there were not enough data points to allow updated modelling of these indicators.

## Discussion

Although progress has been made, there are still major hurdles to overcome for these six indicators to be useful and valuable global health metrics. This study has shown that availability is generally poor; only one indicator (workforce) had data from more than half of the WHO member states, and two (protection against catastrophic and impoverishing expenditure) had virtually no data. Comparability is likewise limited, with many countries and organizations having differing definitions of indicators and their components. Utility is subjective; however, the authors were in agreement that the utility of some of the indicators is limited, in particular access to timely essential surgery. The lack of precise definitions for each indicator and its components also hampers their utility. Indicators are most useful when considered together and not in isolation. The authors' main recommendations are summarized in *Table* 3.

**Table 3:** Recommendations by indicator

	Recommendations
Data collection, reporting and indicator review	Data should be requested of Ministries of Health, collected by the WHO, and made public through the Global Health Observatory and shared to the World Bank World Development Indicators through a formal data‐sharing agreement Data should be compiled and presented every 2 years as a report to the World Health Assembly to facilitate tracking of process Indicators should be reviewed regularly by an international group of experts Countries should encourage improved data collection and use as part of National Surgical, Obstetric and Anaesthesia Plans
Indicator 1: access to timely essential surgery	The indicator should be renamed to Geographic Access to Surgical Facilities Definition and method of verification of surgically capable facility should be refined in a consultative process; e.g. facilities that have performed bellwether procedures in past 3 months as verified in logbooks 2h travel distance calculations should be refined to take into account local context
Indicator 2: specialist surgical workforce density	Definition of specialist surgical providers should be kept and aligned with WHO National Health Workforce Accounts Possibility of collecting data on non‐specialist/non‐physician providers should be evaluated The need for nationally appropriate workforce targets should be emphasized Global targets should be reviewed through a consultative process
Indicator 3: surgical volume	Basic reporting on total number of operations performed in an operating theatre should be encouraged Identifying a representative sample of operations with relatively consistent indications and a more homogeneous demographic to complement the total volume indicator should be evaluated using a consultative process
Indicator 4: perioperative mortality	Collection and use of data at facility level should be encouraged Next steps should be clarified through a consultative process, possibly taking advantage of the above‐mentioned representative sampling of operations to make assessment more meaningful The Commission's target of 80% of countries tracking perioperative mortality rate by 2020 should be endorsed
Indicator 5–6: protection against impoverishing and catastrophic expenditure	Surgery should be integrated into and aligned with a broader research agenda for financial risk protection in healthcare A robust and feasible methodology for collecting out‐of‐pocket cost of surgery should be developed and tested; exploring new and existing tools (Demographic and Health Surveys and Multiple Indicator Cluster Surveys) As there is significant overlap, only one of the two indicators should be selected for reporting

Most low‐ and middle‐income countries are not reaching targets for workforce or number of operations done. Whether the targets are set too high is a necessary point for review. There were too few data points to allow more comprehensive conclusions about 2h access, in‐hospital perioperative mortality, or protection from impoverishing and catastrophic expenditure.

The access to timely essential surgery indicator, as stated, does not truly reflect whether patients can access services, as geographical proximity is just one dimension of access. Nevertheless, it is easily measurable and does provide some top‐level indication of access. For countries with data, most people appear to be within 2h of a facility able to provide the bellwether procedures. Two studies^32^, ^33^ using similar methodology, but without taking into account the capacity to provide bellwether procedures, found that, from 47 and 48 countries across Africa respectively, 71–92 per cent of the population were within 2h of a hospital. However, using methodologies that measure local travel time to facilities more precisely, other studies have found considerably lower proportions of the population within 2h of a facility. The current methods of estimating travel time to facilities may not reflect on‐the‐ground reality^34^. Geographic Information System methods do not account for lack of, or breakdowns in, transport, the impact of road traffic, seasonal effects on travel speeds, or referral patterns between hospitals providing appropriate care. Without validation, whether ability to provide bellwether procedures reflected these operations could not be assessed. In the maternal health community, emergency obstetric and neonatal care capability is ascertained by logbook review^35^, and facility capacity is assessed in terms of ability to serve the patients in their respective catchment area^36^, ^37^, ^38^, ^39^. Such methodologies for capturing true service provision for surgery are feasible but would require greater country‐level investment in, and commitment to, primary data collection. Until more commitment is secured, the authors recommend renaming this indicator geographic access to surgical facilities.

The workforce indicator was the most well defined and readily available, perhaps reflective of the fact that countries have been tracking workforce for a number of years^17^. Number of surgeons has been proposed as a component of measuring the Sustainable Development Goals^10^, and surgical workforce has been included in the WHO National Health Workforce Accounts handbook^40^. However, this study shows that this indicator also faces challenges in its comparability and hence utility. For example, subspecialties included in the definition of surgeon are highly variable, and some databases include trainees or non‐physician providers along with specialists. It was also recognized that using the current Commission definition of this indicator under‐represents the workforce contributing surgical care^41^, ^42^, ^43^, ^44^, ^45^, ^46^, ^47^. For the purposes of comparability and benchmarking, a more uniform definition is needed^40^.

More countries with data on volume of surgery were found (72) than reported previously (56^48^ and 66^18^), but this still represents only two‐fifths of the world's population. This study highlights issues with comparability and utility, noting that volume alone may not reflect the ability to meet country need. The proposed target of 5000 operations per 100 000 population remains subject to manipulation, as it could be achieved by doing many simple procedures, rather than addressing actual population need. Again, potential solutions can be found by looking to the emergency obstetric and neonatal care community, where estimates of population need for emergency obstetrics in the past 3 months are compared with logbooks to estimate the met need^49^. In surgery, given the vast array of conditions that require surgical care, such calculations may be more complex, but have been done^50^. The estimated global volume of surgery for 2015 was lower than the previously published estimate for 2012 (although confidence intervals overlapped). This is likely explained by the exclusion of data from before 2010 and data where nationwide rates were extrapolated from subnational data.

Perioperative mortality is probably the most widely adopted indicator for surgical outcomes, so the inability to identify national data was troubling. Concerns have been voiced previously about the use of this indicator to punish or reward individuals or facilities, producing incentives to selectively treat low‐risk conditions. Similar arguments around the unintended consequences of using perioperative mortality were put forward when individual surgeons were asked to report their mortality data in the UK and USA^51^, ^52^. However, such reporting has become a routine and accepted component of surgical monitoring. Although this metric could provide an indication of quality when appropriately risk‐stratified and adjusted, without this it cannot be used for comparative purposes. Research studies have shown that it is possible to determine risk^53^ by adjusting for a relatively small number of co‐variables^54^, so it could be possible to refine this metric to improve comparability and utility. The recent African Surgical Outcomes Study^55^ and the GlobalSurg studies^53^, ^56^ showed that large international collaborative research efforts can feasibly collect mortality information^57^. It is hoped that there will be more acceptance of collecting perioperative mortality data, particularly if they are used transparently to improve care.

Although the proportion of the population protected from impoverishing and catastrophic expenditure has been modelled previously for 186 countries^20^, no primary national‐level data were found. Some primary subnational data have enabled estimation of these indicators^20^, ^58^, ^59^ but, without country‐level primary data, comparability and utility of this indicator remain low. Primary data could be obtained relatively simply by collecting information on out‐of‐pocket cost of surgery through interviewing samples of patients who have undergone surgery (exit interviews) and using nationally representative sampling strategies; household income data are likely to be collected as part of monitoring for the broader universal health coverage financing agenda. However, to use this metric to monitor surgery as a component of universal health coverage, it is essential to account for people who need but do not access surgery, and to ascertain whether costs are the limitation. More rigorous epidemiological studies would enable calculation of these indicators and improvement of their availability. That potentially life‐saving surgery results in an expenditure which is usually large and unexpected makes it especially relevant for inclusion in universal health coverage initiatives.

This study has important limitations. The data collection interval was 2010–2016, and publication of the Commission report in 2015 and other global initiatives to improve access to safe affordable surgical care in that year were an incentive to collect data. Indeed, data collection is ongoing (frequently driven by professional societies and researchers rather than governments and multilateral organizations) and, in some instances, new data have been published since data collection for this study ended^34^, ^60^, ^61^. New data are not always nationally representative, and have increased availability only marginally; challenges to comparability and utility also remain. Although it is useful that data collection is continuing, this study provides important learning for these increased efforts.

Another limitation of this study is that data quality was not assessed by the authors. Data collected from official sources and publications were assumed to be correct as is standard practice for global health indicators; however, it is possible that, if validated through primary research, some data points might differ. Given the large number of individual data points (which would total 1164 data points per year if all 6 indicators were collected in all countries), it would be impossible to review data quality at the global level; instead, quality assurance mechanisms may be needed at national or regional levels. Finally, this study has mainly addressed factors related to the indicators themselves, rather than their uptake and use for global, national or regional policy and planning purposes. However, the availability, comparability and utility of indicators are crucial to their use, and they frequently underpin National Surgical, Obstetric and Anaesthesia Plans^62^, ^63^, ^64^, ^65^.

In this study, key gaps and opportunities have been identified for further development of the six indicators. The authors propose that an independent international expert group be created to review and update the indicators. Such a process would help improve the availability and quality of data on surgical and anaesthesia care worldwide, a crucial tool to improve access for the billions who lack it today.

Because surgery as a health service is both diverse and complex, a standard set of well defined, representative procedures could be used across many of the indicators to improve collectability and comparability. The concept is similar to a market basket for calculating a consumer price index; it could be adjustable, based on population needs and technical advances, capture frequent health service purchases (both in terms of costs as well as types of operation performed), and be used to compare health commodities (relative proportions of procedures as well as surgical outcomes) across countries and settings. This would expand the bellwether procedures list to add more precision to the collection of surgical information, and would allow improved assessment of specific indicators, such as financial risk protection, perioperative mortality and volume. It would also be more useful for health system planning around surgical services. Defining such a surgical basket that has relevance across countries, or clusters of countries in groups with similar income, and for which indicators can be captured, requires further work and discussion.

Despite multiple calls for more data collection around surgery^2^, ^4^, ^8^, ^9^, none of the indicators is being collected routinely and reported to the WHO. Instead, academics, societies and non‐governmental organizations have been compiling and providing data on surgical and anaesthesia care. To be sustainable, data collection needs to be valued, and driven by ministries and multilateral health organizations, and supported by professional societies, academics and individual providers. With improved definitions, collection and aggregation, these indicators can play an even greater role in improving access to safe, affordable and timely surgical, obstetric and anaesthesia care.

## Supporting information


**Fig. S1.** Relationship between observed operations and total health expenditure per capita, 72 Member States of the World Health Organization, 2015
**Table S1.** Inclusion hierarchy for selecting values by country
**Table S2.** Health system indicators used for multiple imputation
**Table S3.** Inclusion hierarchy for selecting values by country
**Table S4.** Spearman correlation between surgical volume and country level variables in 72 countries with available surgical volume data
**Table S5.** Proportion of population within 2 hours of a surgical facility in 19countries with available data
**Table S6.** Number and proportion of countries included in analysis of Indicator 2
**Table S7.** Surgical workforce databases identified
**Table S8.** Surgical workforce definitions used
**Table S9.** Median specialist surgical workforce per 100,000 people, by category and in total, by WHO region and World Bank Income region (excluding estimated values)
**Table S10.** Estimated 2015 specialist surgical workforce in 1,000 s, by category and in total, by WHO region and World Bank Income region (including estimated values)
**Table S11.** Specialist surgical workforce for 166 WHO member states with available data, including data sources
**Table S12.** Estimated 2015 specialist surgical workforce density per 100,000 for 40 WHO member states with incomplete surgical workforce data, based on multiple imputation
**Table S13.** Number and proportion of countries included in analysis of Indicator 3, by WHO Region and World Bank Income Category
**Table S14.** Volume of surgery databases identified
**Table S15.** Volume of surgery definitions found
**Table S16.** Median surgical volume per 100,000 people by WHO region and World Bank Income region (excluding modelled data)
**Table S17.** Estimated surgical volume in 1,000s, by WHO region and World Bank Income region (including modelled data)
**Table S18.** Surgical volume for 72 WHO member states with available data, including data sources
**Table S19.** Estimated surgical volume in 2015 for 122 WHO member states without available data
**Table S20.** Countries with available POMR data, definition used and data source
**Table S21.** Reported data on cost of surgery for the three Bellwether Procedures, in US$, compared to estimated cost of caesarean section from WHO‐CHOICE* as reported by Shrime et al.^30, 31^
