# Supplementary material for: Evaluating the collection, comparability and findings of six global surgery indicators
Source: Br J Surg. 2018 Dec 20;106(2):e138–50. doi: 10.1002/bjs.11061 (PMC6790969; doi:10.1002/bjs.11061)
Supplement: Supplementary file 1 — Fig. S1. Relationship between observed operations and total health expenditure per capita, 72 Member States of the World Health Organization, 2015 Table S1. Inclusion hierarchy for selecting values by country Table S2. Health system indicators used for multiple imputation Table S3. Inclusion hierarchy for selecting values by country Table S4. Spearman correlation between surgical volume and country level variables in 72 countries with available surgical volume data Table S5. Proportion of population within 2 hours of a surgical facility in 19countries with available data Table S6. Number and proportion of countries included in analysis of Indicator 2 Table S7. Surgical workforce databases identified Table S8. Surgical workforce definitions used Table S9. Median specialist surgical workforce per 100,000 people, by category and in total, by WHO region and World Bank Income region (excluding estimated values) Table S10. Estimated 2015 specialist surgical workforce in 1,000 s, by category and in total, by WHO region and World Bank Income region (including estimated values) Table S11. Specialist surgical workforce for 166 WHO member states with available data, including data sources Table S12. Estimated 2015 specialist surgical workforce density per 100,000 for 40 WHO member states with incomplete surgical workforce data, based on multiple imputation Table S13. Number and proportion of countries included in analysis of Indicator 3, by WHO Region and World Bank Income Category Table S14. Volume of surgery databases identified Table S15. Volume of surgery definitions found Table S16. Median surgical volume per 100,000 people by WHO region and World Bank Income region (excluding modelled data) Table S17. Estimated surgical volume in 1,000s, by WHO region and World Bank Income region (including modelled data) Table S18. Surgical volume for 72 WHO member states with available data, including data sources Table S19. Estimated surgical volume in 2015 for 122 WHO member states w [file BJS-106-e138-s001.docx]

**BJS11061**

# Evaluating the collection, comparability, and findings of six global surgery indicators

H. Holmer, A. Bekele, L. Hagander, E. M. Harrison, P. Kamali, J. S. Ng-Kamstra, M. A. Khan, L. Knowlton, A. J. M. Leather, I. H. Marks, J. G. Meara, M. G. Shrime, M. Smith, K. Søreide, T. G. Weiser and J. Davies

Table of Contents

Methods 3

Indicator 2. Specialist surgical workforce density 3

Indicator 3. Surgical volume 3

Results 4

Indicator 1. Access to timely essential surgery 4

Indicator 2. Specialist surgical workforce density 5

Indicator 3. Surgical volume 15

Indicator 4. Perioperative mortality 22

Indicator 5 and 6. Protection against impoverishing and catastrophic expenditure 23

References 24

# Methods

## Indicator 2. Specialist surgical workforce density

**Table S1** Inclusion hierarchy for selecting values by country

| 1. Direct government contact or report 2. Professional society contact or report 3. WHO/OECD/Eurostat databases 4. Scientific publication 5. Other reports |
| --- |

**Table S2** Health system indicators used for multiple imputation

| Births attended by skilled health staff (% of total)  Corruption Perception Index  Fertility rate, total (births per woman)  GNI per capita, Atlas method (current US$)  Health expenditure per capita (current US$)  Immunization, measles (% of children ages 12-23 months)  Improved water source (% of population with access)  Labour force, female (% of total labour force)  Maternal mortality ratio (modelled estimate, per 100,000 live births) | Mortality rate, infant (per 1,000 live births)  Mortality rate, under-5 (per 1,000 live births)  Nurses and midwives (per 1,000 people)  Out-of-pocket health expenditure (% of total expenditure on health)  Physicians (per 1,000 people)  Population growth (annual %)  Urban population growth (annual %) |
| --- | --- |

##

## Indicator 3. Surgical volume

**Table S3** Inclusion hierarchy for selecting values by country

| 1. Direct government contact or report 2. Published data 3. European Healthcare for All Database (Inpatient only) |
| --- |

**Table S4** Spearman correlation between surgical volume and country level variables in 72 countries with available surgical volume data

|  | **Spearman’s r** | **P-value** |
| --- | --- | --- |
| Total population | -0.156 | 0.192 |
| Life expectancy | 0.574 | <0.001 |
| Percent urbanization | 0.647 | <0.001 |
| GDP per capita (current US$) | 0.704 | <0.001 |
| Total health expenditure per capita | 0.724 | <0.001 |

# Results

## Indicator 1. Access to timely essential surgery

**Table S5** Proportion of population within 2 hours of a surgical facility in 19 countries with available data

| **Country** | **Value** | **Year** | **Source** | **Income category** |
| --- | --- | --- | --- | --- |
| Andorra | 100% | 2015 | Ministry of health | High income |
| Austria | 100% | 2015 | Ministry of health | High income |
| Belgium | 100% | 2016 | Ministry of health | High income |
| Cyprus | 100% | 2016 | Ministry of health | High income |
| Latvia | 100% | 2016 | Ministry of health | High income |
| Lithuania | 100% | 2016 | Ministry of health | High income |
| Monaco | 100% | 2015 | Ministry of health | High income |
| St. Vincent and Grenadines | 100% | 2015 | Ministry of health | Upper middle income |
| Sri Lanka | 100% | 2015 | Ministry of health | Lower middle income |
| Finland | 99% | 2015 | Ministry of health | High income |
| Sweden | 99% | 2016 | Ministry of health | High income |
| Belize | 95% | 2015 | Ministry of health | Upper middle income |
| Iceland | 94% | 2015 | Ministry of health | High income |
| Netherlands | 92% | 2016 | Ministry of health | High income |
| Morocco | 92% | 2016 | Ministry of health | Lower middle income |
| Zambia | 85% | 2016 | Esquivel et al. 2016^1^ | Lower middle income |
| Mongolia | 84% | 2015 | Mongolian National University of Medical Sciences | Lower middle income |
| Seychelles | 84% | 2015 | Ministry of health | High income |
| Vanuatu | 66% | 2016 | Young et al. 2016^2^ | Lower middle income |

## Indicator 2. Specialist surgical workforce density

**Table S6** Number and proportion of countries included in analysis of Indicator 2

|  | **Had data**  *# of countries (%)* | **Did not have data**  *# of countries (%)* | **Total**  *# of countries (%)* |
| --- | --- | --- | --- |
| **WHO Region** |  |  |  |
| African Region | 35 (74.5%) | 12 (25.5%) | 47 |
| Eastern Mediterranean Region | 14 (66.7%) | 7 (33.3%) | 21 |
| European Region | 53 (100.0%) | 0 (0.0%) | 53 |
| Region of the Americas | 31 (88.6%) | 4 (11.4%) | 35 |
| South-East Asia Region | 8 (72.7%) | 3 (27.3%) | 11 |
| Western Pacific Region | 25 (92.6%) | 2 (7.4%) | 27 |
|  |  |  |  |
| **World Bank Income Category** |  |  |  |
| High income | 49 (89.1%) | 6 (10.9%) | 55 |
| Upper middle income | 50 (87.7%) | 7 (12.3%) | 57 |
| Lower middle income | 39 (81.3%) | 9 (18.8%) | 48 |
| Low income | 28 (82.4%) | 6 (17.6%) | 34 |
|  |  |  |  |
| **Total** | 166 (85.6%) | 28 (14.4%) | 194 |
| By population (millions, 2015) | 5 410 (74.0%) | 1 904 (26.0%) | 7 315 |

Based on WHO Regions and World Bank Income Categories from the 2015 WHO World Health Statistics Report; and 2015 population from the World Bank World DataBank^3^ (except for Niue, Cook Islands and Eritrea where populations were retrieved from the WHO Global Health Observatory Data Repository)^4^

**Table S7** Surgical workforce databases identified

| **Database** |
| --- |
| Eurostat^5^  Holmer et al. (2015)^6^  OECD.Stat database^7^  WHO European database on human and technical resources for health^8^  WHO European Health for All database^9^  WHO Global Health Observatory^10^  World Bank World Development Indicators^3^ |

**Table S8** Surgical workforce definitions used

| **Database** | **Definition** |
| --- | --- |
| Lancet Commission on Global Surgery^11^ | Number of specialist surgical, anaesthetic, and obstetric physicians who are working |
| European database on human and technical resources for health^8, 9^  European Health for All database | Surgical specialists are doctors who specialise in the use of surgical techniques to treat disorders and diseases.  Inclusion  - General surgery  - Neurological surgery  - Plastic surgery  - Orthopaedic surgery  - Ophthalmology  - Urology  - Other types of surgery  - Anaesthesiology  - Intensive care  - Accident and emergency medicine  - Medical interns or residents training in these specialties  Exclusion  - Dental surgery  - Oral and maxillofacial surgery  Obstetricians specialise in pregnancy and childbirth. Gynaecologists are concerned with the functions and diseases specific to women and girls, especially those affecting the reproductive system.  Inclusion  - Child/paediatric gynaecology  - Reproduction medicine  - Genetics  - Medical interns or residents specialising in obstetrics and gynaecology |
| WHO Global Health Observatory^10^ and Holmer et al. (2015)^6^ | Number of licensed qualified surgeons actively working  Number of licensed qualified obstetricians actively working  Number of licensed qualified anaesthesiologists actively working |

**Table S9** Median specialist surgical workforce per 100,000 people, by category and in total, by WHO region and World Bank Income region (excluding estimated values)

|  | **Surgeons**  *Median*  *[25^th^-75^th^ percentile]* | **Obstetricians**  *Median*  *[25^th^-75^th^ percentile]* | **Anaesthesiologists**  *Median*  *[25^th^-75^th^ percentile]* | **Total SOAs**  *Median*  *[25^th^-75^th^ percentile]* |
| --- | --- | --- | --- | --- |
| **WHO Region** |  |  |  |  |
| African Region | 0.4 [0.2 – 0.9] | 0.2 [0.1 – 0.8] | 0.1 [0.0 – 0.3] | 1.0 [0.5 – 2.1] |
| Eastern Mediterranean Region | 2.3 [1.3 – 7.4] | 2.8 [1.3 – 4.1] | 1.5 [0.5 – 5.3] | 5.6 [3.6 – 14.4] |
| European Region | 42.0 [31.7 – 56.2] | 17.7 [13.3 – 21.0] | 17.1 [13.2 – 21.6] | 78.2 [60.5 – 105.2] |
| Region of the Americas | 11.0 [5.5 – 19.5] | 6.8 [4.6 – 9.7] | 5.4 [2.1 – 8.6] | 22.4 [12.4 – 36.1] |
| South-East Asia Region | 0.9 [0.7 – 1.5] | 1.0 [0.6 – 2.1] | 0.6 [0.5 – 0.7] | 2.8 [2.3 – 3.5] |
| Western Pacific Region | 6.9 [1.9 – 16.7] | 4.8 [1.4 – 9.3] | 3.6 [0.9 – 6.7] | 14.5 [4.5 – 37.3] |
|  |  |  |  |  |
| **World Bank Income Category** |  |  |  |  |
| High income | 40.5 [18.9 – 52.1] | 14.5 [9.3 – 18.6] | 16.3 [9.7 – 21.4] | 67.9 [48.6 – 89.5] |
| Upper middle income | 11.0 [5.2 – 30.4] | 7.3 [3.8 – 12.7] | 4.6 [2.0 – 9.8] | 23.8 [11.4 – 55.3] |
| Lower middle income | 1.8 [0.7 – 9.2] | 1.7 [0.7 – 6.3] | 0.9 [0.3 – 2.4] | 3.6 [1.6 – 15.1] |
| Low income | 0.4 [0.2 – 0.7] | 0.2 [0.1 – 0.8] | 0.1 [0.0 – 0.3] | 0.7 [0.4 – 1.7] |
|  |  |  |  |  |
| **Total** | 9.9 [1.1 – 32.9] | 6.5 [1.1 – 15.3] | 4.4 [0.6 – 12.7] | 22.3 [3.4 – 60.5] |

**Table S10** Estimated 2015 specialist surgical workforce in 1,000s, by category and in total, by WHO region and World Bank Income region (including estimated values)

|  | **Surgeons** | | **Obstetricians** | | **Anaesthesiologists** | | **SOAs** | | **% of population** |
| --- | --- | --- | --- | --- | --- | --- | --- | --- | --- |
|  | *1000s*  *[25^th^ – 75^th^ percentile]* | *% of total* | *1000s*  *[25^th^ – 75^th^ percentile]* | *% of total* | *1000s [25^th^ – 75^th^ percentile]* | *% of total* | *1000s [25^th^ – 75^th^ percentile]* | *% of total* |  |
| **WHO Region** |  |  |  |  |  |  |  |  |  |
| African Region | 14.1 [12.3 – 19.2] | 1% | 6.1 [5.0 – 9.4] | 1% | 3.8 [3.1 – 5.8] | 1% | 24.1 [20.4 – 34.4] | 1% | 14% |
| Eastern Mediterranean Region | 46.5 [40.1 – 58.5] | 4% | 30.4 [27.8 – 35.1] | 5% | 17.7 [14.7 – 22.3] | 5% | 94.6 [82.6 – 116.0] | 5% | 9% |
| European Region | 371.3 [359.4 – 380.4] | 34% | 156.3 [156.3 – 156.3] | 27% | 170.4 [167.4 – 175.1] | 47% | 697.9 [683.1 – 711.8] | 34% | 13% |
| Region of the Americas | 222.1 [218.4 – 227.5] | 20% | 99.7 [98.0 – 103.0] | 17% | 83.2 [82.4 – 84.9] | 23% | 405.0 [398.9 – 415.4] | 20% | 14% |
| South-East Asia Region | 86.7 [22.9 – 188.2] | 8% | 59.7 [19.8 – 107.6] | 10% | 14.1 [4.6 – 43.9] | 4% | 160.5 [47.3 – 339.7] | 8% | 26% |
| Western Pacific Region | 356.4 [354.2 – 361.5] | 32% | 224.5 [222.6 – 226.4] | 39% | 76.0 [75.8 – 76.6] | 21% | 656.9 [652.6 – 664.5] | 32% | 25% |
|  |  |  |  |  |  |  |  |  |  |
| **World Bank Income Category** |  |  |  |  |  |  |  |  |  |
| High income | 440.3 [437.2 – 446.1] | 40% | 176.3 [175.2 – 178.3] | 31% | 204.3 [202.4 – 207.3] | 56% | 820.9 [814.8 – 831.7] | 40% | 18% |
| Upper middle income | 490.5 [483.1 – 500.0] | 45% | 284.6 [282.1 – 289.1] | 49% | 116.1 [114.2 – 119.1] | 32% | 891.2 [879.5 – 908.2] | 44% | 34% |
| Lower middle income | 158.3 [79.7 – 279.0] | 14% | 110.4 [67.3 – 164.0] | 19% | 41.4 [28.3 – 78.4] | 11% | 310.1 [175.3 – 521.4] | 15% | 37% |
| Low income | 8.0 [7.3 – 10.2] | 1% | 5.4 [5.0 – 6.4] | 1% | 3.3 [3.1 – 3.9] | 1% | 16.7 [15.3 – 20.5] | 1% | 12% |
|  |  |  |  |  |  |  |  |  |  |
| **Total** | 1 097.1  [1 007.3 – 1 235.3] | 100% | 576.7  [529.6 – 637.8] | 100% | 365.1  [348.0 – 408.6] | 100% | 2 038.9  [1 884.9 – 2 281.8] | 100% | 100% |

**Table S11** Specialist surgical workforce for 166 WHO member states with available data, including data sources

| **Country** | **Year** | **Surgeons**  *Total*  *(per 100,000)* | **Obstetricians**  *Total*  *(per 100,000)* | **Anaesthesiologists**  *Total*  *(per 100,000)* | **SOAs**  *Total*  *(per 100,000)* | **Population** *(millions)* | **Source** |
| --- | --- | --- | --- | --- | --- | --- | --- |
| Afghanistan | 2010 |  |  | 8 (0.03) |  | 29 | Dubowitz et al (2010)^12^ |
| Albania | 2013 |  | 191 (6.6) |  |  | 3 | WHO European Health For All Database |
| Algeria | 2013 | 3204 (8.4) | 599 (1.6) | 825 (2.2) | 4628 (12.1) | 38 | Statistiques Sanitaires, Ministère de la Santé de la Population et de la Réforme Hospitalière |
| Andorra | 2014 | 20 (25.2) | 14 (17.7) | 10 (12.6) | 44 (55.5) | 0.08 | Andorra Ministry of Health |
| Antigua and Barbuda | 2014 | 5 (5.1) | 6 (6.1) | 2 (2.0) | 13 (13.1) | 0.1 | Antigua and Barbuda Ministry of Health |
| Argentina | 2014 |  |  | 4500 (10.5) |  | 43 | Argentinean Federation of Anaesthesiologists |
| Armenia | 2015 | 1745 (59.8) | 903 (31.0) | 338 (11.6) | 2986 (102.4) | 3 | Republic of Armenia Ministry of Health (2016) Personal communication |
| Australia | 2015 | 11750 (49.3) | 1473 (6.2) | 4037 (16.9) | 17260 (72.4) | 24 | Australian Government Department of Health (2016) Personal communication |
| Austria | 2015 | 4945 (57.3)* | 1861 (21.6) | 2691 (31.2) | 9497 (110.0) | 9 | Eurostat |
| Azerbaijan | 2014 |  | 1913 (20.1) |  |  | 10 | WHO European Health For All Database |
| Bahrain | 2015 | 101 (7.4) | 47 (3.4) | 49 (3.6) | 197 (14.4) | 1 | Ministry of Health (2016) Human Resources – Ministry of Health |
| Bangladesh | 2012 | 2615 (1.7) | 850 (0.5) | 1200 (0.8) | 4665 (3.0) | 156 | Lebrun et al (2010)^13^ |
| Barbados | 2014 | 18 (6.4) | 12 (4.2) | 19 (6.7) | 49 (17.3) | 0.3 | Barbados Ministry of Health |
| Belarus | 2013 | 6428 (67.9) | 2590 (27.4) | 2466 (26.1) | 11484 (121.3) | 9 | Public Health in the Republic of Belarus, National – Statistical Committee of the Republic of Belarus |
| Belgium | 2015 | 4744 (42.1) | 1640 (14.5) | 2435 (21.6) | 8819 (78.2) | 11 | Ministry of Health National Statistical Committee |
| Belize | 2014 | 23 (6.5) | 4 (1.1) | 10 (2.8) | 37 (10.5) | 0 | Belize Ministry of Health |
| Benin | 2014 | 81 (0.8) | 90 (0.9) | 20 (0.2) | 191 (1.9) | 10 | College des Jeunes Médecins du Bénin |
| Bhutan | 2014 | 6 (0.8) | 13 (1.7) | 3 (0.4) | 22 (2.8) | 1 | Medical Superintendent, Jigme Dorji Wanchuck National Referral Hospital, Thimpu, Bhutan |
| Bolivia (Plurinational State of) | 2012 | 1807 (17.6) | 500 (4.9) | 1270 (12.4) | 3577 (34.9) | 10 | Lebrun et al (2010)^13^ |
| Bosnia and Herzegovina | 2013 | 578 (16.0) | 211 (5.9) | 158 (4.4) | 947 (26.3) | 4 | Institute for Statistics of Federation of Bosina and Hercegovina Statistical Yearbook 2014 |
| Botswana | 2014 | 40 (1.8) | 18 (0.8) | 18 (0.8) | 76 (3.5) | 2 | Botswana Ministry of Health |
| Brazil | 2014 | 40808 (20.0) | 18149 (8.9) | 11492 (5.6) | 70449 (34.5) | 204 | Ministério da Saúde, Departamento de Informática do Sistema Único de Saúde (DATASUS) |
| Bulgaria | 2013 | 5859 (80.6) | 1408 (19.4) | 1511 (20.8) | 8778 (120.8) | 7 | National Statistical Institute, Exhaustive annual survey |
| Burkina Faso | 2014 | 36 (0.2) | 10 (0.1) | 58 (0.3) | 104 (0.6) | 18 | Burkina Faso Ministry of Health |
| Burundi | 2016 | 19 (0.2) |  |  |  | 11 | O'Flynn et al. 2016^14^ |
| Cabo Verde | 2011 | 57 (11.2) | 12 (2.4) | 11 (2.2) | 80 (15.7) | 1 | Relatório estatístico 2011, Ministry of Health |
| Cameroon | 2013 | 83 (0.6) | 151 (1.0) | 22 (0.1) | 256 (1.7) | 15 | Society of Gynecologists and Obstetricians of Cameroon, Médécins du Cameroun |
| Canada | 2016 | 7717 (21.3) | 2098 (5.8) | 3274 (9.0) | 13089 (36.1) | 36 | Canadian Medical Association (2016) Canadian specialty profiles |
| Central African Republic | 2014 | 9 (0.2) | 8 (0.2) | 0 (0.0) | 17 (0.4) | 5 | Système Nationale d'Information de Santé; Sassara Sarl et ACCESS Sarl |
| Chad | 2013 | 18 (0.1) | 13 (0.1) | 2 (0.02) | 33 (0.3) | 13 | Chad Surgical Society |
| Chile | 2012 | 6502 (37.6) | 2117 (12.2) | 1703 (9.8) | 10322 (59.6) | 17 | Ministeria de Salud, Médicos Especialistas del sector, por sexo, registrados en la Superintendencia de Salud. Chile, año 2012 |
| China | 2012 | 293021 (21.7) | 194634 (14.4) | 53471 (4.0) | 541126 (40.1) | 1351 | National Health and Family Planning Commission of People’s Republic of China |
| Colombia | 2015 | 6126 (12.7) | 1995 (4.1) | 2672 (5.5) | 10793 (22.4) | 48 | Government of Colombia Ministry of Health and Social Protection (2016) Personal Communication |
| Congo | 2013 | 7 (0.1) | 3 (0.1) | 0 (0.0) | 10 (0.2) | 5 | Congo-Brazzaville Surgical Society |
| Cook Islands | 2014 | 1 (4.8) | 1 (4.8) | 1 (4.8) | 3 (14.4) | 0.02 | Cook Islands Ministry of Health workforce plan 2014-2020 |
| Costa Rica | 2014 | 406 (8.5) | 375 (7.9) | 280 (5.9) | 1061 (22.3) | 5 | College of Physicians & Surgeons of Costa Rica |
| Côte d’Ivoire | 2014 | 318 (1.4) | 238 (1.1) | 80 (0.4) | 636 (2.8) | 23 | Conseil National de l'Ordre des Médecins de Côte d'Ivoire; Société Ivoirienne d'Anesthesie Réanimation |
| Croatia | 2015 | 1756 (41.8)* | 782 (18.6) | 877 (20.9) | 3415 (81.2) | 4 | Eurostat |
| Cuba | 2016 | 6271 (54.6) | 2656 (23.1) | 1414 (12.3) | 10341 (90.1) | 11 | Ministerio de Salud Pública, Direccion de Registros Medicos y Estadisticas de Salud, Annuario Estadístico de Salud 2016 |
| Cyprus | 2015 | 629 (54.2)* | 162 (14.0) | 176 (15.2) | 967 (83.3) | 1 | Eurostat |
| Czech Republic | 2013 | 7629 (72.6) | 2512 (23.9) | 1940 (18.5) | 13092 (114.9) | 11 | Institute of Health Information and Statistics of the Czech Republic; Registry of Physicians, Dentists and Pharmacists |
| Democratic Republic of the Congo | 2013 | 54 (0.08) | 39 (0.05) | 32 (0.04) | 125 (0.18) | 71 | Longombe (2014)^15^ |
| Denmark | 2014 | 2283 (40.5)* | 581 (10.3) | 1029 (18.2) | 3893 (69.0) | 6 | Eurostat |
| Djibouti | 2013 | 17 (1.9) | 13 (1.4) | 9 (1.0) | 39 (4.3) | 1 | Djibouti Ministry of Health |
| Dominica | 2014 | 2 (2.7) | 4 (5.5) | 2 (2.7) | 8 (11.0) | 0.07 | Dominica Ministry of Health |
| Ecuador | 2014 | 5461 (34.3) | 2016 (12.7) | 1996 (12.6) | 9473 (59.6) | 16 | Ecuador Ministry of Health |
| Egypt | 2014 | 23966 (26.1) | 15363 (16.7) | 5533 (6.0) | 44862 (48.9) | 92 | Egyptian Medical Syndicate |
| Estonia | 2016 | 704 (53.5) | 256 (19.5) | 281 (21.4) | 1241 (94.3) | 1 | Estonia National Institute for Health Development, Health Statistics and Health Research Database |
| Ethiopia | 2014 | 349 (0.4) | 147 (0.2) | 44 (0.05) | 540 (0.6) | 97 | Ethiopia Ministry of Health |
| Fiji | 2014 | 16 (1.8) | 12 (1.4) | 9 (1.0) | 37 (4.2) | 1 | Fiji Medical Council |
| Finland | 2014 | 4081 (74.7) | 1000 (18.3) | 933 (17.1) | 6014 (110.1) | 5 | Finnish Medical Association |
| France | 2013 | 18793 (28.5) | 10247 (15.5) | 7076 (10.7) | 36116 (54.7) | 66 | Conseil National de l'Ordre des Médecins |
| Gambia | 2011 | 10 (0.6) | 8 (0.5) | 1 (0.1) | 19 (1.1) | 2 | Gambia Medical and Dental Council; Directorate of Health Promotion and Education, The Gambia Ministry of Health and Social Welfare |
| Georgia | 2016 | 2234 (60.1) | 1790 (48.1) |  |  | 4 | National Statistics Office of Georgia |
| Germany | 2015 | 49887 (61.1)* | 17124 (21.0) | 21214 (26.0) | 88225 (108.0) | 82 | Eurostat |
| Greece | 2015 | 12094 (111.8)* | 3376 (31.2) | 2227 (20.6) | 17697 (163.5) | 11 | Eurostat |
| Grenada | 2014 | 3 (2.8) | 7 (6.6) | 2 (1.9) | 12 (11.3) | 0.1 | Grenada Ministry of Health |
| Guatemala | 2014 | 201 (1.3) | 188 (1.2) | 155 (1.0) | 544 (3.4) | 16 | Professional Societies; Surgery only covers general surgery |
| Guyana | 2014 | 45 (5.9) | 13 (1.7) | 9 (1.2) | 67 (8.8) | 1 | Medical Council of Guyana |
| Haiti | 2014 | 115 (1.1) | 300 (2.8) | 60 (0.6) | 475 (4.5) | 11 | Dr. Gerald Lerebours, via Tú M. TRẦN |
| Honduras | 2014 | 444 (5.0) | 498 (5.7) | 147 (1.7) | 1089 (12.4) | 9 | Colegio Médico de Honduras |
| Hungary | 2010 |  | 1220 (12.2) |  |  | 10 | OECD |
| Iceland | 2015 | 160 (48.4)* | 44 (13.3) | 57 (17.2) | 261 (78.9) | 0.3 | Eurostat |
| Iran (Islamic Republic of) | 2014 | 1272 (1.6) | 1197 (1.5) | 1421 (1.8) | 3890 (5.0) | 78 | Ministry of Health Office of Information and Statistics |
| Iraq | 2013 | 1071 (3.2) | 974 (2.9) | 488 (1.4) | 2533 (7.5) | 34 | Iraq Ministry of Health, Annual Statistical Report 2013 |
| Ireland | 2016 | 1192 (25.1)* | 369 (7.8) | 748 (15.7) | 2309 (48.6) | 5 | Eurostat |
| Israel | 2013 | 3161 (39.2) | 636 (7.9) | 1049 (13.0) | 4846 (60.1) | 8 | Israel Ministry of Health |
| Italy | 2016 | 41407 (68.3)* | 12180 (20.1) | 13046 (21.5) | 66633 (109.9) | 61 | Eurostat |
| Jamaica | 2014 | 183 (6.4) | 99 (3.5) | 40 (1.4) | 322 (11.3) | 3 | Medical Council of Jamaica, University of the West Indies Department of Surgery, Radiology and Intensive Care |
| Japan | 2013 | 21275 (16.7) | 10412 (8.2) | 8140 (6.4) | 39827 (31.3) | 127 | Japanese Board of Medical Specialities; Japanese Ministry of Health |
| Kazakhstan | 2015 | 5567 (31.7) | 4928 (28.1) | 2337 (13.3) | 12832 (73.1) | 18 | Ministry of Health and Social Development of the Republic of Kazakhstan (2016) Activity of Health and Health Sources of the Population of the Republic of Kazakhstan in 2015 (Report in Kazakh) |
| Kenya | 2014 | 316 (0.7) | 357 (0.8) | 129 (0.3) | 802 (1.7) | 46 | Kenya Medical Practitioners and Dentists Board |
| Kiribati | 2014 | 1 (0.9) | 2 (1.8) | 2 (1.8) | 5 (4.5) | 0.1 | Medical Council Register at the Kiribati Ministry of Health and Medical Services |
| Kyrgyzstan | 2013 | 1868 (32.7) | 994 (17.4) | 592 (10.4) | 3454 (60.4) | 6 | Kyrgyz Republic Ministry of Health |
| Lao People's Democratic Republic | 2014 | 65 (1.0) | 66 (1.0) | 60 (0.9) | 191 (2.9) | 7 | University of health Sciences of Lao PDR; Lao Association of Obstetric Gynecology; Lao Society of Anesthesiologists |
| Latvia | 2016 | 937 (47.8) | 423 (21.6) | 394 (20.1) | 1754 (89.5) | 2 | Ministry of Health of the Republic of Latvia (2016) Personal communication |
| Lebanon | 2014 | 2547 (45.5) | 941 (16.8) | 486 (8.7) | 3974 (70.9) | 6 | Lebanese Order of Physicians (Excludes North Lebanon Order of Physicians) |
| Lesotho | 2014 | 4 (0.2) | 3 (0.1) | 5 (0.2) | 12 (0.6) | 2 | Queen Mamohato Nemorial Hospital, Maseru, Lesotho |
| Lithuania | 2015 | 1624 (55.9)* | 714 (24.6) | 891 (30.7) | 3229 (111.2) | 3 | Eurostat |
| Luxembourg | 2016 | 280 (48.1)* | 95 (16.3) | 95 (16.3) | 470 (80.8) | 1 | Eurostat |
| Madagascar | 2016 | 94 (0.4) | 14 (0.1) | 26 (0.1) | 134 (0.5) | 25 | Bruno et al. (2017)^16^ |
| Malawi | 2014 | 61 (0.4) | 7 (0.06) | 11 (0.1) | 79 (0.5) | 17 | Medical Council of Malawi |
| Malaysia | 2011 | 1967 (6.9) | 836 (2.9) | 684 (2.4) | 3487 (12.2) | 29 | Malaysia National Healthcare Establishment & Workforce Statistics 2011 |
| Maldives | 2014 | 6 (1.5) | 13 (3.2) | 2 (0.5) | 21 (5.1) | 0.4 | Maldives Medical Council |
| Mali | 2014 | 151 (0.9) |  | 21 (0.1) |  | 17 | Société D’Anesthésie Réanimation Médecine D’urgence Du Mali |
| Malta | 2016 | 204 (46.6) | 67 (15.3) | 102 (23.3) | 373 (85.3) | 0.4 | Eurostat |
| Marshall Islands | 2011 | 6 (11.4) | 2 (3.8) | 1 (1.9) | 9 (17.1) | 0.05 | Ministry of Health Republic of the Marshall Islands Annual Report |
| Mauritius | 2014 | 88 (7.0) | 79 (6.3) | 67 (5.3) | 234 (18.6) | 1 | Medical College of Mauritius |
| Mexico | 2012 | 25900 (21.4) | 14455 (12.0) | 8750 (7.2) | 49105 (40.6) | 121 | Federación Mexicana de Colegios de Obstetricia y Ginecologia |
| Micronesia (Federated States of) | 2014 | 11 (10.6) | 11 (10.6) | 7 (6.7) | 29 (27.9) | 0.1 | Micronesia Anesthesia Society Database |
| Monaco | 2014 | 42 (110.1) | 8 (21.0) | 23 (60.3) | 73 (191.4) | 0.04 | Monaco Ministry of Health |
| Mongolia | 2014 | 409 (14.0) | 677 (23.2) | 238 (8.1) | 1324 (45.3) | 3 | National Center of Health Development, Mongolia Ministry of Health |
| Montenegro | 2016 | 205 (32.9) | 111 (17.8) | 83 (13.3) | 399 (64.1) | 1 | Eurostat |
| Morocco | 2016 | 822 (2.3) | 284 (0.8) | 180 (0.5) | 1286 (3.6) | 35 | Ministry of Health of Morocco |
| Mozambique | 2013 | 186 (0.7) | 63 (0.2) | 28 (0.1) | 277 (1.0) | 26 | Mozambique Ministry of Health |
| Myanmar | 2013 | 478 (0.9) | 466 (0.9) | 310 (0.6) | 1254 (2.4) | 51 | Myanmar Ministry of Health, Department of Medical Science |
| Namibia | 2014 | 80 (3.4) | 27 (1.1) | 26 (1.1) | 35 (5.6) | 2 | Medical and Dental Council of Namibia, Register for Medical Practitioners and Specialists |
| Nauru | 2014 | 1 (8.4) | 1 (8.4) | 1 (8.4) | 3 (25.3) | 0.01 | Report of the Royal Australasian College of Surgeons |
| Nepal | 2014 | 254 (0.9) | 336 (1.2) | 193 (0.7) | 783 (2.8) | 28 | Society of Surgeons of Nepal; Society of Anesthesiologists of Nepal; Nepal Society of Obstetricians and Gynaecologists |
| Netherlands | 2015 | 4672 (27.6)* | 1571 (9.3) | 2053 (12.1) | 8296 (49.0) | 17 | Eurostat |
| New Zealand | 2015 | 856 (18.6) | 689 (15.0) | 264 (5.7) | 1809 (39.4) | 5 | Medical Council of New Zealand |
| Nicaragua | 2014 | 524 (8.7) | 269 (4.5) | 157 (2.6) | 950 (15.8) | 6 | Ministerio De Salud, Division General De Recursos Humanos, Distribución De Medicos Especialistas Por Departamento Y Municipio |
| Niger | 2014 | 31 (0.2) | 42 (0.2) | 6 (0.03) | 79 (0.4) | 19 | Direction des Ressources Humaines/Ministère de la Santé Publique du Niger |
| Nigeria | 2014 | 1827 (1.0) | 454 (0.3) | 144 (0.1) | 2425 (1.4) | 176 | Medical and Dental Council of Nigeria |
| Niue | 2014 | 1 (62.5) | 1 (62.5) | 0 (0.0) | 2 (125.0) | 0.002 | Niue Health Department Data |
| Norway | 2013 | 2012 (39.6) | 546 (10.7) | 793 (15.6) | 3351 (66.0) | 5 | Statistics Norway; Register-based statistics on employment of health-care personnel |
| Oman | 2015 | 679 (16.2) | 222 (5.3) | 246 (5.9) | 1147 (27.3) | 4 | Ministry of Health (2016) Annual Health Report 2015 |
| Pakistan | 2012 | 2239 (1.3) | 4951 (2.8) | 2722 (1.5) | 9912 (5.6) | 178 | Pakistan Medical and Dental Council |
| Palau | 2012 | 1 (4.8) | 1 (4.8) | 1 (4.8) | 3 (14.5) | 0.02 | Strengthening Specialised Clinical Services in the Pacific Program (2012) Mapping of Clinician's Profiles in 14 Pacific Island Countries |
| Panama | 2014 | 423 (10.8) | 391 (10.0) | 209 (5.4) | 1023 (26.2) | 4 | Panama Ministry of Health, Dirección De Recursos Humanos, Dpto. De Estadística De Planificación |
| Papua New Guinea | 2014 | 36 (0.5) | 18 (0.2) | 24 (0.3) | 78 (1.0) | 8 | Papua New Guinea Obstetrics and Gynaecology Society |
| Paraguay | 2014 | 356 (5.4) | 429 (6.5) | 157 (2.4) | 942 (14.4) | 7 | Paraguay Ministry of Health |
| Peru | 2014 | 8804 (28.4) | 2679 (8.6) | 1382 (4.5) | 12865 (41.5) | 31 | Peruvian Society of Surgeons |
| Philippines | 2014 | 2500 (2.5) | 3362 (3.4) | 3611 (3.6) | 9473 (9.5) | 100 | Philippine College of Surgeons; Philippine Obstetrics and Gynecologists Society; Philippine Society of Anesthesiologists |
| Poland | 2015 | 15671 (41.3)* | 5046 (13.3) | 5062 (13.3) | 25779 (67.9) | 38 | Eurostat |
| Portugal | 2015 | 5364 (51.8)* | 1726 (16.7) | 1827 (17.6) | 8917 (86.1) | 10 | Eurostat |
| Qatar | 2011 | 67 (3.4) | 81 (4.1) | 128 (6.6) | 276 (14.1) | 2 | The Hamad Medical Corporation, Annual Health Report (2011) |
| Republic of Korea | 2014 | 15792 (31.1) | 5463 (10.8) | 3771 (7.4) | 25026 (49.3) | 51 | National Health Insurance Service (NHIS) and Health Insurance Review and Assessment Service (HIRA) |
| Republic of Moldova | 2013 | 563 (15.8) | 641 (18.0) | 553 (15.5) | 1757 (49.4) | 4 | National Centre of Management in Health |
| Romania | 2015 | 7451 (37.6)* | 2523 (12.7) | 2021 (10.2) | 11995 (60.5) | 20 | Eurostat |
| Russian Federation | 2014 | 23842 (16.6) | 28894 (20.1) | 38050 (26.5) | 90786 (63.1) | 144 | Russian Federation Ministry of Health |
| Rwanda | 2014 | 42 (0.4) | 25 (0.2) | 15 (0.1) | 82 (0.7) | 11 | Rwanda Medical and Dental Council and Rwanda Professional Societies |
| Saint Kitts and Nevis | 2014 | 6 (11.2) | 8 (14.9) | 3 (5.6) | 17 (31.6) | 0.05 | Saint Kitts and Nevis Ministry of Health, Chief Medical Officer |
| Saint Lucia | 2014 | 20 (11.3) | 15 (8.5) | 7 (4.0) | 42 (23.8) | 0.2 | Saint Lucia Medical and Dental Council |
| Saint Vincent and the Grenadines | 2014 | 4 (3.7) | 8 (7.3) | 2 (1.8) | 14 (12.8) | 0.1 | Saint Vincent and the Grenadines, Ministry of Health, Wellness and the Environment |
| Samoa | 2014 | 5 (2.6) | 5 (2.6) | 4 (2.1) | 14 (7.3) | 0.2 | Samoa National Health Serivces |
| San Marino | 2014 | 17 (52.1) | 7 (21.4) | 12 (36.7) | 36 (110.2) | 0.03 | Istituto per la Sicurezza Sociale: Ordine dei medici e degli Odontoiatri |
| Senegal | 2011 | 38 (0.3) | 57 (0.4) | 42 (0.3) | 137 (1.0) | 13 | Senegal Ministry of Health |
| Serbia | 2014 | 2359 (33.1)* | 1292 (18.1) | 950 (13.3) | 4601 (64.5) | 7 | Eurostat |
| Seychelles | 2014 | 22 (24.1) | 6 (6.6) | 4 (4.4) | 32 (35.0) | 0.09 | Seychelles Ministry of health |
| Sierra Leone | 2012 | 5 (0.1) | 3 (0.04) | 1 (0.01) | 9 (0.1) | 7 | Ministry of Health and Sanitation |
| Singapore | 2013 | 1019 (18.9) | 304 (5.6) | 375 (6.9) | 1698 (31.4) | 5 | Singapore Medical Council |
| Slovakia | 2014 | 1001 (18.5) | 961 (17.7) | 1136 (21.0) | 3098 (57.2) | 5 | Slovak Medical Chamber, Register of Health Professionals |
| Slovenia | 2015 | 716 (34.7)* | 333 (16.1) | 330 (16.0) | 1379 (66.8) | 2 | Eurostat |
| Solomon Islands | 2012 | 9 (1.6) | 4 (0.7) | 5 (0.9) | 18 (3.3) | 1 | Strengthening Specialised Clinical Services in the Pacific Program (2012) Mapping of Clinician's Profiles in 14 Pacific Island Countries |
| Somalia | 2014 | 15 (0.1) | 5 (0.04) | 1 (0.007) | 21 (0.2) | 14 | Ministry of Health |
| South Africa | 2014 | 3453 (6.3) | 1631 (3.0) | 1143 (2.1) | 6227 (11.4) | 55 | Health Professions Council of South Africa |
| South Sudan | 2013 | 15 (0.1) | 17 (0.2) | 1 (0.009) | 33 (0.3) | 11 | Ministry of Health, Republic of South Sudan, Dr. Richard Lino Loro Lako personal communication |
| Spain | 2015 | 24079 (51.8)* | 5570 (12.0) | 11093 (23.9) | 40742 (87.7) | 46 | Eurostat |
| Sri Lanka | 2014 | 169 (0.8) | 127 (0.6) | 107 (0.5) | 375 (1.9) | 21 | Sri Lanka Ministry of Health |
| Sudan | 2014 | 300 (0.8) | 500 (1.3) | 250 (0.7) | 1050 (2.8) | 38 | Sudan Professional Societies |
| Swaziland | 2010 |  |  | 2 (0.2) |  | 1 | Dubowitz et al. (2010)^12^ |
| Sweden | 2015 | 8070 (82.4) | 1364 (13.9) | 1650 (16.8) | 11084 (113.1) | 10 | Swedish National Board of Health and Welfare |
| Switzerland | 2016 | 4632 (55.3)* | 1773 (21.2) | 2048 (24.5) | 8453 (101.0) | 8 | Eurostat |
| Tajikistan | 2014 | 1309 (15.7) | 1486 (17.8) | 645 (7.7) | 3440 (41.1) | 8 | Statistics and Medical Information Centre, Tajikistan Ministry of Health and Social Protection of the Population |
| Thailand | 2014 | 4284 (6.3) | 3154 (4.6) | 1523 (2.2) | 8961 (13.1) | 68 | The Medical Council of Thailand |
| The former Yugoslav Republic of Macedonia | 2015 | 657 (31.6) | 391 (18.8) | 225 (10.8) | 1273 (61.2) | 2 | Eurostat |
| Timor-Leste | 2014 | 5 (0.4) | 1 (0.1) | 1 (0.1) | 7 (0.6) | 1 | Timor-Leste Ministry of Health |
| Togo | 2014 | 20 (0.3) | 10 (0.1) | 6 (0.1) | 36 (0.5) | 7 | Clinique Medico Chirurgicale, Togo |
| Tonga | 2014 | 3 (2.8) | 1 (0.9) | 1 (0.9) | 5 (4.7) | 0.1 | Ministry of Health, Tonga |
| Trinidad and Tobago | 2014 | 247 (18.2) | 99 (7.3) | 75 (5.5) | 421 (31.1) | 1 | Medical Board of Trinidad and Tobago |
| Turkey | 2015 | 24268 (31.0)* | 6956 (8.9) | 6206 (7.9) | 37430 (47.8) | 78 | Eurostat |
| Turkmenistan | 2014 | 1572 (28.8) | 756 (13.8) | 694 (12.7) | 3022 (55.3) | 5 | Turkmenistan Ministry of Health |
| Tuvalu | 2012 | 1 (9.3) | 1 (9.3) | 2 (18.6) | 4 (37.3) | 0.01 | Strengthening Specialised Clinical Services in the Pacific Program (2012) Mapping of Clinician's Profiles in 14 Pacific Island Countries |
| Uganda | 2012 | 204 (0.6) | 124 (0.3) | 17 (0.05) | 345 (1.0) | 36 | Linden et al. (2012)^17^ |
| Ukraine | 2014 |  | 11061 (24.4) |  |  | 45 | WHO European Health For All Database |
| United Kingdom | 2016 | 27537 (42.0)* | 7533 (11.5) | 15884 (24.2) | 50954 (77.7) | 66 | Eurostat |
| United Republic of Tanzania | 2010 | 100 (0.2) | 50 (0.1) | 6 (0.01) | 156 (0.3) | 46 | Ministry of Health of Tanzania |
| United States of America | 2015 | 93009 (29.0) | 41481 (12.9) | 41351 (12.9) | 175841 (54.8) | 321 | American Medical Association (2016) Number of People per Active Physician by Specialty, 2015 |
| Uruguay | 2014 | 400 (11.7) | 600 (17.5) | 330 (9.7) | 1330 (38.9) | 3 | Uruguay Professional Society |
| Uzbekistan | 2014 |  | 5255 (17.1) |  |  | 31 | WHO European Health for All Database |
| Vanuatu | 2014 | 5 (1.9) | 2 (0.8) | 2 (0.8) | 9 (3.5) | 0.3 | Vanuatu Ministry of Health |
| Venezuela (Bolivarian Republic of) | 2014 | 3411 (11.1) | 2154 (7.0) |  |  | 31 | Venezuelan Surgery Society; Venezuelan Obstetrics and Gynaecology Society |
| Yemen | 2014 | 104 (0.4) | 93 (0.4) | 15 (0.1) | 212 (0.8) | 26 | Yemeni Board of Medical Specialization |
| Zambia | 2012 | 97 (0.7) | 46 (0.3) | 13 (0.1) | 156 (1.1) | 15 | The 2012 Annual Report; Health Professions Council of Zambia |
| Zimbabwe | 2014 | 80 (0.5) | 63 (0.4) | 48 (0.3) | 191 (1.2) | 15 | The Medical and Dental Practitioners Council of Zimbabwe 2014 statistics |

* Calculated from “Surgical group of specialists” by subtracting anaesthesiologists and emergency physicians

**Table S12** Estimated 2015 specialist surgical workforce density per 100,000 for 40 WHO member states with incomplete surgical workforce data, based on multiple imputation

| **Country** | **Surgeons**  **Median**  *[25^th^ – 75^th^ percentile]* | **Obstetricians**  **Median**  *[25^th^ – 75^th^ percentile]* | **Anaesthesiologists**  **Median**  *[25^th^ – 75^th^ percentile]* |
| --- | --- | --- | --- |
| Afghanistan | 0.99 [0.46 – 3.23] | 0.38 [0.14 – 1.08] | – |
| Albania | 7.17 [5.04 – 11.42] | – | 1.18 [0.39 – 2.77] |
| Angola | 1.63 [0.56 – 5.15] | 0.83 [0.25 – 2.85] | 0.91 [0.18 – 2.67] |
| Argentina | 21.44 [15.65 – 29.32] | 9.32 [6.90 – 14.41] | – |
| Azerbaijan | 31.36 [20.68 – 39.22] | – | 13.01 [10.31 – 17.64] |
| Bahamas | 18.63 [7.36 – 32.98] | 7.16 [4.14 – 15.52] | 7.34 [2.66 – 13.09] |
| Brunei Darussalam | 16.10 [9.32 – 31.86] | 3.09 [1.00 – 6.59] | 10.35 [6.39 – 13.32] |
| Burundi | – | 0.35 [0.14 – 1.18] | 0.59 [0.12 – 2.08] |
| Cameroon | 3.11 [0.81 – 7.36] | 1.62 [0.86 – 4.82] | 0.39 [0.13 – 1.83] |
| Comoros | 1.93 [0.52 – 9.18] | 5.79 [2.97 – 10.58] | 0.91 [0.13 – 5.31] |
| Democratic People’s Republic of Korea | 16.10 [6.26 – 31.24] | 7.54 [2.87 – 13.46] | 5.74 [1.81 – 11.01] |
| Dominican Republic | 8.36 [2.75 – 16.03] | 5.57 [2.20 – 11.96] | 1.82 [0.14 – 6.12] |
| El Salvador | 10.71 [2.80 – 28.51] | 7.31 [3.41 – 12.73] | 4.42 [0.75 – 10.35] |
| Equatorial Guinea | 2.50 [0.45 – 8.44] | 4.61 [1.14 – 8.54] | 0.97 [0.13 – 3.61] |
| Eritrea | 7.17 [1.47 – 16.85] | 2.86 [0.83 – 6.20] | 0.91 [0.13 – 3.70] |
| Gabon | 3.37 [0.81 – 8.71] | 1.81 [0.82 – 4.61] | 1.81 [0.16 – 4.95] |
| Georgia | – | – | 20.80 [18.45 – 26.05] |
| Ghana | 2.33 [0.79 – 6.99] | 1.76 [0.60 – 5.79] | 0.69 [0.12 – 2.32] |
| Guinea | 1.81 [0.70 – 7.36] | 0.89 [0.23 – 2.84] | 0.91 [0.19 – 2.39] |
| Guinea-Bissau | 2.50 [0.77 – 6.62] | 0.41 [0.14 – 1.08] | 0.91 [0.13 – 2.27] |
| Hungary | 31.60 [21.40 – 39.22] | – | 12.59 [10.35 – 13.34] |
| India | 4.82 [0.91 – 11.27] | 3.27 [0.83 – 6.27] | 0.54 [0.06 – 2.04] |
| Indonesia | 6.08 [1.22 – 12.70] | 4.61 [1.53 – 7.95] | 1.40 [0.14 – 5.32] |
| Jordan | 17.65 [8.71 – 29.32] | 12.20 [7.66 – 16.43] | 10.60 [7.31 – 13.33] |
| Kuwait | 19.98 [15.24 – 31.03] | 6.60 [4.23 – 9.50] | 12.66 [9.84 – 15.54] |
| Liberia | 1.26 [0.54 – 5.27] | 0.35 [0.15 – 1.35] | 1.81 [0.46 – 2.75] |
| Libya | 11.13 [3.66 – 28.47] | 6.60 [1.55 – 12.29] | 3.78 [0.73 – 8.11] |
| Mali | – | 0.73 [0.15 – 1.73] | – |
| Mauritania | 1.47 [0.44 – 3.45] | 0.34 [0.15 – 1.00] | 0.31 [0.08 – 0.95] |
| Sao Tome and Principe | 1.74 [0.70 – 7.70] | 1.60 [0.35 – 4.87] | 0.96 [0.14 – 3.97] |
| Saudi Arabia | 8.71 [4.53 – 22.29] | 2.93 [0.70 – 7.80] | 4.91 [0.94 – 12.19] |
| Suriname | 5.05 [1.63 – 11.10] | 1.56 [0.78 – 4.26] | 1.06 [0.23 – 3.97] |
| Swaziland | 0.89 [0.37 – 2.60] | 0.31 [0.13 – 1.14] | – |
| Syrian Arab Republic | 16.37 [9.17 – 33.40] | 7.31 [3.81 – 15.28] | 6.73 [4.19 – 13.09] |
| Tunisia | 5.04 [0.90 – 8.46] | 4.35 [2.92 – 6.75] | 1.40 [0.31 – 2.15] |
| Ukraine | 42.08 [28.98 – 54.29] | – | 15.16 [12.12 – 21.36] |
| United Arab Emirates | 28.42 [11.17 – 37.56] | 2.86 [0.56 – 6.07] | 11.47 [6.03 – 16.45] |
| Uzbekistan | 28.00 [15.65 – 34.34] | – | 13.02 [9.84 – 16.97] |
| Venezuela [Bolivarian Republic of] | – | – | 1.90 [0.77 – 4.49] |
| Viet Nam | 2.99 [0.70 – 8.36] | 3.46 [1.48 – 5.53] | 0.29 [0.06 – 0.92] |

‘–‘ shown where primary data were available

## Indicator 3. Surgical volume

**Table S13** Number and proportion of countries included in analysis of Indicator 3, by WHO Region and World Bank Income Category

|  | **Had data**  *# of countries (%)* | **Did not have data**  *# of countries (%)* | **Total**  *# of countries (%)* |
| --- | --- | --- | --- |
| **WHO Region** |  |  |  |
| African Region | 8 (17%) | 39 (83%) | 47 |
| Eastern Mediterranean Region | 7 (33%) | 14 (67%) | 21 |
| European Region | 36 (68%) | 17 (32%) | 53 |
| Region of the Americas | 11 (31%) | 24 (69%) | 35 |
| South-East Asia Region | 4 (36%) | 7 (64%) | 11 |
| Western Pacific Region | 6 (22%) | 21 (78%) | 27 |
|  |  |  |  |
| **World Bank Income Category** |  |  |  |
| High income | 27 (49%) | 28 (51%) | 55 |
| Upper middle income | 20 (35%) | 37 (65%) | 57 |
| Lower middle income | 18 (38%) | 30 (63%) | 48 |
| Low income | 7 (21%) | 27 (79%) | 34 |
|  |  |  |  |
| **Total** | 72 (37%) | 122 (63%) | 194 |
| By population (millions, 2015) | 2 837 (39%) | 4 478 (61%) | 7 315 |

Based on WHO Regions and World Bank Income Categories from the 2015 WHO World Health Statistics Report; and 2015 population from the World Bank World DataBank^3^ (except for Niue, Cook Islands and Eritrea where populations were retrieved from the WHO Global Health Observatory Data Repository)^4^

**Table S14** Volume of surgery databases identified

| **Database** |
| --- |
| Eurostat^18^  OECD.Stat database^19^  Weiser et al. (2016)^20^  WHO European Health for All database^21^  World Bank World Development Indicators^3^ |

**Table S15** Volume of surgery definitions found

| **Source** | **Procedure definition** |
| --- | --- |
| Eurostat | Total, inpatient, outpatient and day case  List of procedures |
| WHO EURO  Health For All Database | Inpatient only  All procedures (national definition) |
| OECD Database | Total (inpatient and outpatient)  List of procedures |
| World Bank WDI | Procedures per 100,000  National definitions |
| Weiser et al. 2016^20^ | Total procedures  National definitions |
| Government contacts and reports | Various definitions |

**Table S16** Median surgical volume per 100,000 people by WHO region and World Bank Income region (excluding modelled data)

|  | **Surgical volume**  *per 100,000 [25^th^ – 75^th^ percentile]* |
| --- | --- |
| **WHO Region** |  |
| African Region | 343 [261 – 1 024] |
| Eastern Mediterranean Region | 2 452 [1 182 – 2 960] |
| European Region | 6 753 [4 430 – 10 540] |
| Region of the Americas | 3 005 [1 744 – 4 800] |
| South-East Asia Region | 1 184 [553 – 1 924] |
| Western Pacific Region | 4 334 [2 901 – 6 085] |
|  |  |
| **World Bank Income Category** |  |
| High income | 7 579 [5 014 – 10 891] |
| Upper middle income | 3 375 [2 034 – 12 352] |
| Lower middle income | 2 445 [1 012 – 4 731] |
| Low income | 328 [231 – 513] |
|  |  |
| **Total** | 4 171 [1 892 – 8 526] |

Notes: Total health expenditure adjusted to United States dollars (US$) for the year 2015. The adjusted cross validation was r^2^ = 0.607. Inflexion points correspond with adjusted total health expenditure per capita; the first inflexion point is US$ 262 and the second inflection point is US$ 939.

**Fig. S1** Relationship between observed operations and total health expenditure per capita, 72 Member States of the World Health Organization, 2015

**Table S17** Estimated surgical volume in 1,000s, by WHO region and World Bank Income region (including modelled data)

|  | **Surgical volume**  *Millions [95% Confidence Interval]* | **Percent of all operations, globally** | **Percent of population** *(2015)* |
| --- | --- | --- | --- |
| **WHO Region** |  |  |  |
| African Region | 10.0 [7.3 – 14.0] | 4% | 14% |
| Eastern Mediterranean Region | 11.9 [9.1 – 15.8] | 4% | 9% |
| European Region | 72.6 [63.8 – 86.2] | 27% | 13% |
| Region of the Americas | 92.7 [70.6 – 135.8] | 35% | 14% |
| South-East Asia Region | 16.6 [12.0 – 23.2] | 6% | 26% |
| Western Pacific Region | 62.3 [57.4 – 69.4] | 23% | 25% |
|  |  |  |  |
| **World Bank Income Category** |  |  |  |
| High income | 117.2 [84.7 – 176.0] | 44% | 18% |
| Upper middle income | 115.9 [110.0 – 124.4] | 44% | 34% |
| Lower middle income | 29.5 [22.9 – 38.7] | 11% | 37% |
| Low income | 3.6 [2.5 – 5.3] | 1% | 12% |
|  |  |  |  |
| **Total** | 266.1 [220.1 – 344.4] | 100% | 100% |

**Table S18** Surgical volume for 72 WHO member states with available data, including data sources

| **Country** | **Year** | **Surgical volume**  *Total (per 100,000)* | **2015 population** | **Source** |
| --- | --- | --- | --- | --- |
| Albania | 2013 | 55 038 (1 901) | 2 880 703 | European Health for All Database (HFA-DB) |
| Andorra | 2014 | 4 150 (5 238) | 78 014 | Andorra Ministry of Health |
| Armenia | 2015 | 126 671 (4 343) | 2 916 950 | Ministry of Health, Dept of Foreign Affairs |
| Australia | 2014 | 6 874 535 (29 302) | 23 850 784 | Australian Department of Health, International Strategies Branch |
| Austria | 2012 | 1 178 284 (13 977) | 8 633 169 | EuroREACH 2013 report* |
| Azerbaijan | 2014 | 177 809 (1 865) | 9 649 341 | European Health for All Database (HFA-DB) |
| Bahrain | 2012 | 51 992 (3 999) | 1 371 855 | Health statistics 2012, Manama: Bahraini Ministry of Health* |
| Belarus | 2013 | 1 745 524 (18 440) | 9 489 616 | National Statistical Committee |
| Belgium | 2014 | 2 354 948 (21 009) | 11 274 196 | Belgium Ministry of Health |
| Belize | 2014 | 6 906 (1 964) | 359 288 | Belize Ministry of Health |
| Bhutan | 2015 | 20 445 (2 597) | 787 386 | Annual Helath Bulletin 2016* |
| Bolivia (Plurinational State of) | 2010 | 228 622 (2 305) | 10 724 705 | LeBrun et al. 2012^22^ |
| Brazil | 2015 | 28 163 578 (13 674) | 205 962 108 | DATASUS, Brazilian Medical Demography, Federal Council of Medicine |
| Bulgaria | 2010 | 509 080 (6 884) | 7 177 991 | European Health for All Database (HFA-DB) |
| Burkina Faso | 2012 | 54 379 (328) | 18 110 624 | Ministry of Health of Burkina Faso, Annuaire statistique 2012* |
| Chad | 2012 | 6 593 (52) | 14 009 413 | Ministère de la Santé Publique, Annuaire des statistiques sanitaires du Tchad 2012* |
| China | 2012 | 36 902 542 (2 732) | 1 371 220 000 | National Health and Family Planning Commission of People’s Republic of China |
| Colombia | 2015 | 13 207 282 (27 385) | 48 228 697 | Ministry of Health |
| Costa Rica | 2014 | 178 213 (3 746) | 4 807 852 | Anuario Estadistico 2014 |
| Cuba | 2012 | 539 528 (4 740) | 11 461 432 | Anuario estadístico de salud* |
| Cyprus | 2014 | 35 262 (3 060) | 1 160 985 | Ministry of Health |
| Czech Republic | 2012 | 658 811 (6 268) | 10 546 059 | Institute of Health Information and Statistics of the Czech Republic; 2012* |
| Denmark | 2010 | 564 130 (10 169) | 5 683 483 | European Health for All Database (HFA-DB) |
| Ecuador | 2014 | 242 266 (1 523) | 16 144 368 | Ministry of Health |
| Estonia | 2014 | 127 633 (9 709) | 1 315 407 | European Health for All Database (HFA-DB) |
| Finland | 2010 | 621 731 (11 592) | 5 479 531 | European Health for All Database (HFA-DB) |
| Georgia | 2012 | 189 478 (4 954) | 3 717 100 | Georgian Ministry of Labour Health and Social Affairs, Health statistical database, national centre for control and public health* |
| Guatemala | 2012 | 231 288 (1 515) | 16 252 429 | Ministerio de Salud Pública y Asistencia Social, Sistema de Información Gerencial de Salud |
| Hungary | 2010 | 1 382 738 (13 827) | 9 843 028 | European Health for All Database (HFA-DB) |
| Ireland | 2010 | 139 219 (3 053) | 4 646 554 | European Health for All Database (HFA-DB) |
| Israel | 2012 | 400 808 (5 067) | 8 380 100 | Head of Division of Health Information, Israeli Ministry of Health* |
| Jordan | 2015 | 226 506 (2 473) | 9 159 302 | Ministry of Health |
| Kazakhstan | 2015 | 782 245 (4 459) | 17 544 126 | European Health for All Database (HFA-DB) |
| Kyrgyzstan | 2015 | 181 390 (3 045) | 5 956 900 | European Health for All Database (HFA-DB) |
| Latvia | 2015 | 363 941 (18 404) | 1 977 527 | Ministry of Health |
| Lithuania | 2015 | 295 992 (10 189) | 2 904 910 | Ministry of Health |
| Malta | 2012 | 55 501 (13 232) | 431 874 | National Hospitals Information System, Directorate for Health Information & Research, Malta* |
| Mauritius | 2014 | 25 938 (2 057) | 1 262 605 | Ministry of Health |
| Mexico | 2012 | 1 613 405 (1 335) | 125 890 949 | Sistema Nacional de Información en Salud (SINAIS): Secretaría de Salud, Mexico Ministry of Health* |
| Mongolia | 2014 | 153 849 (5 262) | 2 976 877 | Center for Health Development |
| Morocco | 2015 | 259 731 (746) | 34 803 322 | Ministry of health |
| Myanmar | 2011 | 337 726 (668) | 52 403 669 | Annual hospital statistics report 2010–2011* |
| Nepal | 2011 | 56 768 (208) | 28 656 282 | Nepalese Ministry of Health and Population, 2011 Annual report* |
| New Zealand | 2012 | 280 310 (6 359) | 4 595 700 | New Zealand Ministry of Health, National minimum dataset (NMDS) 2014* |
| Nicaragua | 2010 | 278 874 (4 860) | 6 082 035 | Solis et al. 2013^23^ |
| Niger | 2014 | 48 800 (255) | 19 896 965 | Ministry of Health, public facilities only |
| Oman | 2015 | 102 965 (2 452) | 4 199 810 | MoH report |
| Peru | 2011 | 894 243 (3 005) | 31 376 671 | Peruvian Ministry of Health, Memoria Institucional de Essalud* |
| Portugal | 2011 | 890 965 (8 439) | 10 358 076 | Statistics Portugal* |
| Republic of Korea | 2012 | 1 709 706 (3 406) | 51 014 947 | Main Surgery Statistical Yearbook 2012* |
| Republic of Moldova | 2014 | 174 206 (4 898) | 3 554 108 | European Health for All Database (HFA-DB) |
| Russian Federation | 2013 | 9 502 577 (6 622) | 144 096 870 | European Health for All Database (HFA-DB) |
| Samoa | 2015 | 1 635 (844) | 193 759 | European Health for All Database (HFA-DB) |
| San Marino | 2015 | 1 635 (4 961) | 32 960 | European Health for All Database (HFA-DB) |
| Saudi Arabia | 2012 | 1 002 474 (3 447) | 31 557 144 | Saudi Arabian Ministry of Health* |
| Seychelles | 2012 | 24 152 (27 351) | 93 419 | Seychelles Ministry of health |
| Sierra Leone | 2012 | 24 152 (357) | 7 237 025 | Bolkan et al. 2015^24^ |
| Slovakia | 2012 | 475 111 (8 786) | 5 423 801 | Health statistics yearbook of the Slovak Republic* |
| Slovenia | 2010 | 155 265 (7 579) | 2 063 531 | European Health for All Database (HFA-DB) |
| South Sudan | 2014 | 30 256 (262) | 11 882 136 | Ministry of Health, HMIS Report |
| Spain | 2010 | 4 657 900 (10 000) | 46 444 832 | Ministerio de Sanidad, Servicios Sociales e Igualdad; 2012* |
| Sri Lanka | 2015 | 356 268 (1 699) | 20 966 000 | Health Performance Monitoring Indicators, 2015, Ministry of Health, Nutrition and Indigenous Medicine, Sri Lanka |
| Sweden | 2014 | 1 476 569 (15 228) | 9 799 186 | National Board of Health and Welfare |
| Syrian Arab Republic | 2010 | 339 825 (1 617) | 18 734 987 | Syrian Ministry of Health* |
| Tajikistan | 2015 | 143 426 (1 678) | 8 548 651 | European Health for All Database (HFA-DB) |
| Turkey | 2010 | 8 614 789 (11 911) | 78 271 472 | European Health for All Database (HFA-DB) |
| Turkmenistan | 2015 | 144 970 (2 605) | 5 565 284 | European Health for All Database (HFA-DB) |
| Ukraine | 2011 | 2 439 132 (5 337) | 45 154 029 | European Health for All Database (HFA-DB) |
| United Kingdom | 2010 | 4 507 258 (7 181) | 65 128 861 | European Health for All Database (HFA-DB) |
| Uzbekistan | 2015 | 808 824 (2 584) | 31 298 900 | European Health for All Database (HFA-DB) |
| Yemen | 2014 | 41 272 (157) | 26 916 207 | Ministry of Health |
| Zambia | 2010 | 94 145 (680) | 16 100 587 | Personal communication with Bowman K, Children’s Hospital of Wisconsin, United States of America* |

* Full source presented in Weiser et al. 2016^20^

**Table S19** Estimated surgical volume in 2015 for 122 WHO member states without available data

| **Country** | **2015 population** | **Average imputed no. of operations per 100 000 population per year** | **Expected range of operations in 2015*** |
| --- | --- | --- | --- |
| Afghanistan | 33 736 494 | 692 | 162 689 – 335 455 |
| Algeria | 39 871 528 | 3 823 | 1 043 636 – 2 226 087 |
| Angola | 27 859 305 | 1 349 | 281 186 – 502 531 |
| Antigua and Barbuda | 99 923 | 5 489 | 4 003 – 7 515 |
| Argentina | 43 417 765 | 6 527 | 1 839 489 – 4 366 323 |
| Bahamas | 386 838 | 7 386 | 20 987 – 38 897 |
| Bangladesh | 161 200 886 | 338 | 321 018 – 923 887 |
| Barbados | 284 217 | 6 763 | 13 048 – 28 321 |
| Benin | 10 575 952 | 332 | 20 591 – 59 821 |
| Bosnia and Herzegovina | 3 535 961 | 4 550 | 122 763 – 210 852 |
| Botswana | 2 209 197 | 4 348 | 72 050 – 128 066 |
| Brunei Darussalam | 417 542 | 6 032 | 17 051 – 37 196 |
| Burundi | 10 199 270 | 249 | 13 817 – 46 849 |
| Cambodia | 15 517 635 | 817 | 90 844 – 176 956 |
| Cameroon | 22 834 522 | 738 | 118 744 – 239 221 |
| Canada | 35 832 513 | 9 314 | 2 145 966 – 5 190 074 |
| Cabo Verde | 532 913 | 1 881 | 7 378 – 13 617 |
| Central African Republic | 4 546 100 | 162 | 3 549 – 15 347 |
| Chile | 17 762 681 | 6 682 | 793 868 – 1 774 458 |
| Comoros | 777 424 | 672 | 3 616 – 7 542 |
| Congo | 4 995 648 | 676 | 23 394 – 48 691 |
| Cook Islands | 20 800 | 4 689 | 748 – 1 271 |
| Croatia | 4 203 604 | 6 162 | 171 963 – 390 136 |
| Côte d'Ivoire | 23 108 472 | 896 | 150 252 – 285 049 |
| Democratic Republic of the Congo | 76 196 619 | 198 | 76 805 – 296 423 |
| Djibouti | 927 414 | 979 | 6 663 – 12 371 |
| Dominica | 73 162 | 4 320 | 2 363 – 4 227 |
| Dominican Republic | 10 528 394 | 4 384 | 347 559 – 613 027 |
| Egypt | 93 778 172 | 2 039 | 1 394 578 – 2 620 781 |
| El Salvador | 6 312 478 | 3 773 | 161 215 – 351 931 |
| Equatorial Guinea | 1 175 389 | 3 754 | 29 730 – 65 499 |
| Eritrea | 5 227 800 | 327 | 9 993 – 29 256 |
| Ethiopia | 99 873 033 | 249 | 135 302 – 458 753 |
| Fiji | 892 149 | 2 309 | 14 770 – 28 734 |
| France | 66 624 068 | 9 069 | 4 022 577 – 9 075 440 |
| Gabon | 1 930 175 | 2 654 | 35 879 – 73 156 |
| Gambia | 1 977 590 | 339 | 3 956 – 11 364 |
| Germany | 81 686 611 | 9 354 | 4 884 429 – 11 954 170 |
| Ghana | 27 582 821 | 951 | 191 786 – 358 463 |
| Greece | 10 820 883 | 7 191 | 561 501 – 1 078 362 |
| Grenada | 106 823 | 4 684 | 3 839 – 6 523 |
| Guinea | 12 091 533 | 259 | 17 162 – 57 029 |
| Guinea-Bissau | 1 770 526 | 431 | 4 792 – 12 175 |
| Guyana | 768 514 | 2 453 | 13 383 – 26 544 |
| Haiti | 10 711 061 | 609 | 44 251 – 96 037 |
| Honduras | 8 960 829 | 2 334 | 149 725 – 292 266 |
| Iceland | 330 815 | 9 249 | 19 860 – 47 135 |
| India | 1 309 053 980 | 734 | 6 764 750 – 13 653 941 |
| Indonesia | 258 162 113 | 1 394 | 2 692 486 – 4 811 580 |
| Iran (Islamic Republic of) | 79 360 487 | 4 230 | 2 480 411 – 4 542 793 |
| Iraq | 36 115 649 | 2 008 | 529 939 – 992 232 |
| Italy | 60 730 582 | 8 254 | 3 660 943 – 6 863 530 |
| Jamaica | 2 871 934 | 3 839 | 75 753 – 160 427 |
| Japan | 127 141 000 | 8 909 | 7 705 627 – 16 648 437 |
| Kenya | 47 236 259 | 824 | 279 120 – 542 353 |
| Kiribati | 112 407 | 1 346 | 1 132 – 2 023 |
| Kuwait | 3 935 794 | 6 775 | 181 367 – 392 063 |
| Lao People's Democratic Republic | 6 663 967 | 601 | 27 112 – 59 153 |
| Lebanon | 5 851 479 | 5 444 | 233 753 – 434 140 |
| Lesotho | 2 174 645 | 1 104 | 17 815 – 32 358 |
| Liberia | 4 499 621 | 813 | 26 194 – 51 101 |
| Libya | 6 234 955 | 3 943 | 172 797 – 349 773 |
| Luxembourg | 569 604 | 10 054 | 32 945 – 99 554 |
| Madagascar | 24 234 088 | 212 | 26 598 – 98 869 |
| Malawi | 17 573 607 | 367 | 38 809 – 107 019 |
| Malaysia | 30 723 155 | 4 329 | 995 484 – 1 777 082 |
| Maldives | 418 403 | 6 442 | 17 188 – 42 271 |
| Mali | 17 467 905 | 466 | 52 039 – 127 347 |
| Marshall Islands | 52 994 | 5 811 | 2 153 – 4 404 |
| Mauritania | 4 182 341 | 609 | 17 279 – 37 499 |
| Micronesia (Federated States of) | 104 433 | 4 375 | 3 437 – 6 074 |
| Monaco | 38 307 | 8 663 | 2 328 – 4 731 |
| Montenegro | 622 159 | 4 312 | 20 036 – 35 916 |
| Mozambique | 28 010 691 | 296 | 47 227 – 145 775 |
| Namibia | 2 425 561 | 4 512 | 83 302 – 143 769 |
| Nauru | 12 475 | 6 031 | 509 – 1 111 |
| Netherlands | 16 939 923 | 9 428 | 1 009 930 – 2 525 379 |
| Nigeria | 181 181 744 | 1 192 | 1 610 081 – 2 897 329 |
| Niue | 1 600 | 6 208 | 65 – 151 |
| Norway | 5 190 239 | 10 490 | 293 046 – 1 011 473 |
| Pakistan | 189 380 513 | 413 | 485 387 – 1 260 178 |
| Palau | 21 288 | 7 094 | 1 077 – 2 117 |
| Panama | 3 969 249 | 6 378 | 162 598 – 394 101 |
| Papua New Guinea | 7 919 825 | 920 | 53 063 – 99 979 |
| Paraguay | 6 639 119 | 3 991 | 188 081 – 373 375 |
| Philippines | 101 716 359 | 1 608 | 1 218 758 – 2 195 776 |
| Poland | 37 986 412 | 5 980 | 1 549 862 – 3 329 481 |
| Qatar | 2 481 539 | 7 717 | 142 607 – 257 124 |
| Romania | 19 815 481 | 4 602 | 697 713 – 1 192 002 |
| Rwanda | 11 629 553 | 648 | 51 855 – 109 672 |
| Saint Kitts and Nevis | 54 288 | 6 335 | 2 224 – 5 320 |
| Saint Lucia | 177 206 | 4 780 | 6 504 – 11 030 |
| Saint Vincent and the Grenadines | 109 455 | 3 778 | 2 802 – 6 103 |
| Sao Tome and Principe | 195 553 | 2 087 | 2 968 – 5 612 |
| Senegal | 14 976 994 | 390 | 35 706 – 95 439 |
| Serbia | 7 095 383 | 4 822 | 262 652 – 445 713 |
| Singapore | 5 535 002 | 7 931 | 326 256 – 590 715 |
| Solomon Islands | 587 482 | 1 973 | 8 487 – 15 825 |
| South Africa | 55 291 225 | 4 732 | 2 008 489 – 3 407 583 |
| Sudan | 38 647 803 | 1 968 | 557 221 – 1 038 497 |
| Suriname | 553 208 | 5 182 | 21 606 – 38 036 |
| Swaziland | 1 319 011 | 3 186 | 28 388 – 62 215 |
| Switzerland | 8 282 396 | 11 190 | 448 956 – 1 913 167 |
| Thailand | 68 657 600 | 2 946 | 1 388 656 – 2 946 859 |
| The former Yugoslav Republic of Macedonia | 2 079 308 | 3 845 | 55 016 – 116 176 |
| Timor-Leste | 1 240 977 | 845 | 7 551 – 14 560 |
| Togo | 7 416 802 | 396 | 18 029 – 47 813 |
| Tonga | 106 364 | 3 012 | 2 190 – 4 688 |
| Trinidad and Tobago | 1 360 092 | 6 744 | 62 048 – 135 599 |
| Tunisia | 11 273 661 | 3 579 | 265 715 – 612 804 |
| Tuvalu | 11 001 | 4 587 | 386 – 660 |
| Uganda | 40 144 870 | 513 | 134 809 – 315 225 |
| United Arab Emirates | 9 154 302 | 7 072 | 460 600 – 909 968 |
| United Republic of Tanzania | 53 879 957 | 337 | 106 817 – 307 994 |
| United States of America | 320 896 618 | 11 113 | 17 473 188 – 72 783 456 |
| Uruguay | 3 431 552 | 6 924 | 165 541 – 340 991 |
| Vanuatu | 264 603 | 1 216 | 2 400 – 4 311 |
| Venezuela (Bolivarian Republic of) | 31 155 134 | 6 489 | 1 301 688 – 3 139 470 |
| Viet Nam | 93 571 567 | 1 463 | 1 023 633 – 1 831 503 |
| Zimbabwe | 15 777 451 | 1 151 | 135 084 – 244 015 |

Table excludes North Korea and Somalia due to lack of Health Expenditure data

* Derived from the 95% confidence interval from 300 imputed datasets for each country based on per capita total health expenditure

## Indicator 4. Perioperative mortality

**Table S20** Countries with available POMR data, definition used and data source

| **Country** | **Year of most recent data** | **Income category** | **Definition** | **Source** |
| --- | --- | --- | --- | --- |
| Andorra | 2015 | High income | In-hospital | Statistics department, Andorra Ministry of Finance |
| Australia | 2014-15 | High income | In-hospital | Australian Government Department of Health |
| Belgium | 2014 | High income | In-hospital | Belgium Directorate General Healthcare |
| Brazil | 2016 | Upper middle income | In-hospital | Massenburg et al. 2017^25^ |
| Finland | 2014 | High income | In-hospital | National Institute for Health and Welfare |
| Madagascar | 2016 | Low income | In-hospital | Bruno et al. 2017^16^ |
| Mexico | 2014 | Upper middle income | In-hospital | Urbe-Leitz et al. 2016^26^ |
| Monaco | 2014 | High income | In-hospital | Ministry of Health and Social Affairs, Monaco |
| Spain | 2012 | High income | In-hospital | Ministry of Health, Social Services and Equality |
| Belize | 2014 | Upper middle income | 30-day | Belize Ministry of Health |
| Liberia | 2013 | Low income | 30-day | Knowlton et al. 2013^27^ |
| New Zealand | 2015 | High income | 30-day | Perioperative Mortality in New Zealand: Fourth report of the Perioperative Mortality Review Committee^28^ |
| Sweden | 2015 | High income | 30-day | National Board of Health and Welfare |
| Tonga | 2013 | Upper middle income | 30-day | Ministry of Health, Kingdom of Tonga |
| Tuvalu | 2013 | Upper middle income | 30-day | Princess Margaret Hospital |
| United Kingdom | 2011 | High income | 30-day | Knowing the Risk: A review of the peri-operative care of surgical patients^29^ |
| Armenia | 2016 | Lower middle income | Not defined | Armenia Ministry of Health |
| Belarus | 2015 | Upper middle income | Not defined | National Statistical Committee of the Republic of Belarus |
| Colombia | 2015 | Upper middle income | Not defined | Colombia Ministry of Health and Social Protection |
| Cyprus | 2014 | High income | Not defined | Health Monitoring Unit, Ministry of Health |
| Ecuador | 2014 | Upper middle income | Not defined | Ecuador Ministry of Health |
| Guinea-Bissau | 2016 | Low income | Not defined | Guinea-Bissau Ministry of Health |
| Latvia | 2015 | High income | Not defined | Ministry of Health of the Republic of Latvia |
| Lithuania | 2015 | High income | Not defined | Ministry of Health of the Republic of Lithuania |
| Mongolia | 2014 | Lower middle income | Not defined | Center for Health Development |
| Seychelles | 2014 | High income | Not defined | Ministry of Health Seychelles |
| Solomon Islands | 2014 | Lower middle income | Not defined | Surgical Care in the Solomon Islands: A Road Map for Universal Surgical Care Delivery |
| South Sudan | 2014 | Low income | Not defined | Ministry of Health |

## Indicator 5 and 6. Protection against impoverishing and catastrophic expenditure

**Table S21** Reported data on cost of surgery for the three Bellwether Procedures, in US$, compared to estimated cost of caesarean section from WHO-CHOICE* as reported by Shrime et al.^30, 31^

| **Country** | **Caesarean Section**  *(WHO-CHOICE)* | **Reported** | | | **Year** | **Source** |
| --- | --- | --- | --- | --- | --- | --- |
|  |  | **Caesarean section** | **Laparotomy** | **Open fracture** |  |  |
| Belgium | 803 | 447 | 624 | 389 | 2014 | Ministry of Health |
| Colombia | 203 | 198 – 213 | 155 – 357 | N/A | 2015-16 | Ministry of Health |
| Latvia | 5 131 | 598 | 1090 – 1733 | N/A | 2015 | Ministry of Health |
| Lithuania | 2 014 | 1 089 – 1 219 | 1 106 – 2 321 | 1 079 – 1 180 | 2015 | Ministry of Health |
| Sweden | 1 311 | 7 119 – 7 664 | 12 549 – 19 625 | 9 233 – 11 069 | 2014 | National Board of Health and Welfare |

Currency exchanged to US$ using yearly average exchange rate from the United States Internal Revenue Service (1 US$ = 0.784 Euro in 2014 and 0.932 in 2015, and 7.138 Swedish Krona in 2014),^32^ and X-Rates.com (Colombian Peso; average of monthly averages 2015-16 used; 1 US$ = 2,870.13 Pesos).^33^ Ranges refer to the range of costs provided for primary and tertiary level surgical services; where there is no range, costs were the same across levels.

* WHO-CHOICE (CHOosing Interventions that are Cost-Effective) is a WHO initiative started to guide priority setting in health (http://www.who.int/choice/en/)

# References

1. Esquivel MM, Uribe-Leitz T, Makasa E, Lishimpi K, Mwaba P, Bowman K, et al. Mapping Disparities in Access to Safe, Timely, and Essential Surgical Care in Zambia. *JAMA surgery* 2016;**151**(11): 1064-1069.

2. Young S, Perry WR, Leodoro B, Nosa V, Bissett I, Windsor JA, et al. Challenges and Opportunities in the Provision of Surgical Care in Vanuatu: A Mixed Methods Analysis. *World journal of surgery* 2016;**40**(8): 1865-1873.

3. World Development Indicators. <http://databank.worldbank.org/data/reports.aspx?source=world-development-indicators> [April 1, 2018 2018].

4. Global Health Observatory data repository: Population (1000s). <http://apps.who.int/gho/data/node.main.HWF9?lang=en> [6 May 2018].

5. Eurostat – Physicians by medical speciality (hlth_rs_spec). <http://ec.europa.eu/eurostat/data/database> 23 Oct 2017].

6. Holmer H, Lantz A, Kunjumen T, Finlayson S, Hoyler M, Siyam A, et al. Global distribution of surgeons, anaesthesiologists, and obstetricians. *The Lancet Global health* 2015;**3 Suppl 2**: S9-11.

7. OECD.Stat Health Care Resources: Physicians by categories. <http://stats.oecd.org> [23 Oct 2017 2017].

8. European database on human and technical resources for health (HlthRes-DB) – Surgical group of specialists, total. <https://gateway.euro.who.int/en/datasets/european-database-on-human-and-technical-resources-for-health/> 23 Oct 2017].

9. European Health for All database (HFA-DB) – Number of physicians, surgical group of specialties (PP); Number of physicians, obstetric and gynaecological group of specialties (PP). <https://gateway.euro.who.int/en/datasets/european-health-for-all-database/> 23 Oct 2017].

10. Global Health Observatory data repository: Surgical workforce Reported data by country. <http://apps.who.int/gho/data/node.main.HWF9?lang=en> [6 May 2018].

11. Meara JG, Leather AJ, Hagander L, Alkire BC, Alonso N, Ameh EA, et al. Global Surgery 2030: evidence and solutions for achieving health, welfare, and economic development. *Lancet* 2015;**386**(9993): 569-624.

12. Dubowitz G, Detlefs S, McQueen KA. Global anesthesia workforce crisis: a preliminary survey revealing shortages contributing to undesirable outcomes and unsafe practices. *World journal of surgery* 2010;**34**(3): 438-444.

13. Lebrun DG, Dhar D, Sarkar MI, Imran TM, Kazi SN, McQueen KA. Measuring global surgical disparities: a survey of surgical and anesthesia infrastructure in Bangladesh. *World journal of surgery* 2013;**37**(1): 24-31.

14. O'Flynn E, Andrew J, Hutch A, Kelly C, Jani P, Kakande I, et al. The Specialist Surgeon Workforce in East, Central and Southern Africa: A Situation Analysis. *World journal of surgery* 2016;**40**(11): 2620-2627.

15. Longombe AO. La Cartographie des Medecins Specialistes dans les Quatre Grandes Disciplines Cliniques ainsi que l’Anesthesie-Reanimation en Republique Democratique Du Congo *Great Lakes Medical Review* 2014;**6**(2): 8-12.

16. Bruno E, White MC, Baxter LS, Ravelojaona VA, Rakotoarison HN, Andriamanjato HH, et al. An Evaluation of Preparedness, Delivery and Impact of Surgical and Anesthesia Care in Madagascar: A Framework for a National Surgical Plan. *World journal of surgery* 2017;**41**(5): 1218-1224.

17. Linden AF, Sekidde FS, Galukande M, Knowlton LM, Chackungal S, McQueen KA. Challenges of surgery in developing countries: a survey of surgical and anesthesia capacity in Uganda's public hospitals. *World journal of surgery* 2012;**36**(5): 1056-1065.

18. Surgical operations and procedures performed in hospitals by ICD-9-CM (hlth_co_proc2). <http://ec.europa.eu/eurostat/data/database> 23 Oct 2017].

19. OECD.Stat Health Care Utilisation: Surgical procedures. <http://stats.oecd.org> [23 Oct 2017 2017].

20. Weiser TG, Haynes AB, Molina G, Lipsitz SR, Esquivel MM, Uribe-Leitz T, et al. Size and distribution of the global volume of surgery in 2012. *Bulletin of the World Health Organization* 2016;**94**(3): 201-209f.

21. European Health for All database (HFA-DB) – Total number of inpatient surgical procedures per year. <https://gateway.euro.who.int/en/indicators/hfa_539-6031-total-number-of-inpatient-surgical-procedures-per-year/> 23 Oct 2017].

22. Lebrun DG, Saavedra-Pozo I, Agreda-Flores F, Burdic ML, Notrica MR, McQueen KA. Surgical and anesthesia capacity in Bolivian public hospitals: results from a national hospital survey. *World journal of surgery* 2012;**36**(11): 2559-2566.

23. Solis C, Leon P, Sanchez N, Burdic M, Johnson L, Warren H, et al. Nicaraguan surgical and anesthesia infrastructure: survey of Ministry of Health hospitals. *World journal of surgery* 2013;**37**(9): 2109-2121.

24. Bolkan HA, Von Schreeb J, Samai MM, Bash-Taqi DA, Kamara TB, Salvesen O, et al. Met and unmet needs for surgery in Sierra Leone: A comprehensive, retrospective, countrywide survey from all health care facilities performing operations in 2012. *Surgery* 2015;**157**(6): 992-1001.

25. Massenburg BB, Saluja S, Jenny HE, Raykar NP, Ng-Kamstra J, Guilloux AGA, et al. Assessing the Brazilian surgical system with six surgical indicators: a descriptive and modelling study. *BMJ global health* 2017;**2**(2): e000226.

26. Uribe-Leitz T, Osuna SRR, Esquivel MM, Trejo AC, Garland NY, Cervantes J, et al. Abstract 54.06 The Volume and Outcomes of Surgical Procedures in Mexico in 2014. *Academic Surgical Congress Abstracts* 2016.

27. Knowlton LM, Chackungal S, Dahn B, LeBrun D, Nickerson J, McQueen K. Liberian surgical and anesthesia infrastructure: a survey of county hospitals. *World journal of surgery* 2013;**37**(4): 721-729.

28. Perioperative Mortality Review Committee. Perioperative Mortality in New Zealand: Fourth report of the Perioperative Mortality Review Committee. Wellington, New Zealand; 2015.

29. National Confidential Enquiry into Patient Outcome and Death. Knowing the Risk: A review of the peri-operative care of surgical patients. London, UK; 2011.

30. Shrime MG, Dare A, Alkire BC, Meara JG. A global country-level comparison of the financial burden of surgery. *The British journal of surgery* 2016;**103**(11): 1453-1461.

31. Shrime MG, Dare AJ, Alkire BC, O'Neill K, Meara JG. Catastrophic expenditure to pay for surgery worldwide: a modelling study. *The Lancet Global health* 2015;**3 Suppl 2**: S38-44.

32. Translating foreign currency into U.S. dollars. <https://www.irs.gov/individuals/international-taxpayers/yearly-average-currency-exchange-rates> [19 May 2018].

33. US Dollar per 1 Colombian Peso Monthly average. <www.x-rates.com> [19 May 2018].
